# First computational design using lambda-superstrings and in vivo validation of SARS-CoV-2 vaccine

**DOI:** 10.1038/s41598-022-09615-w

**Published:** 2022-04-19

**Authors:** Luis Martínez, Iker Malaina, David Salcines-Cuevas, Héctor Terán-Navarro, Andrea Zeoli, Santos Alonso, Ildefonso M. De la Fuente, Elena Gonzalez-Lopez, J. Gonzalo Ocejo-Vinyals, Mónica Gozalo-Margüello, Jorge Calvo-Montes, Carmen Alvarez-Dominguez

**Affiliations:** 1 Department of Mathematics, Faculty of Science and Technology, University of the Basque Country, UPV/EHU, 48940 Leioa, Spain; 2 BCAM, Basque Center for Applied Mathematics, 48009 Bilbao, Spain; 3 BioCruces Health Research Institute, Cruces University Hospital, 48903 Barakaldo, Spain; 4 Instituto de Investigación Marqués de Valdecilla (IDIVAL), 39011 Santander, Spain; 5 Department of Genetics, Physical Anthropology and Animal Physiology, Faculty of Science and Technology, University of the Basque Country, UPV/EHU, 48940 Leioa, Spain; 6 María Goyri Building. Animal Biotechnology Center, University of the Basque Country, UPV/EHU, 48940 Leioa, Spain; 7 Servicio de Inmunología, Hospital Universitario Marqués de Valdecilla, 39008 Santander, Spain; 8 Servicio de Microbiología, Hospital Universitario Marqués de Valdecilla, 39008 Santander, Spain; 9 Universidad Internacional de La Rioja, 26006 Logroño, Spain; 10 CIBER Enfermedades Infecciosas, ISCIII, Madrid, Spain; 11 Department of Nutrition, CEBAS-CSIC Institute, Espinardo University Campus, 30100 Murcia, Spain

**Keywords:** Computational biology and bioinformatics, Immunology

## Abstract

Coronavirus disease 2019 (COVID-19) is the greatest threat to global health at the present time, and considerable public and private effort is being devoted to fighting this recently emerged disease. Despite the undoubted advances in the development of vaccines against severe acute respiratory syndrome coronavirus 2 (SARS-CoV-2), the causative agent of COVID-19, uncertainty remains about their future efficacy and the duration of the immunity induced. It is therefore prudent to continue designing and testing vaccines against this pathogen. In this article we computationally designed two candidate vaccines, one monopeptide and one multipeptide, using a technique involving optimizing lambda-superstrings, which was introduced and developed by our research group. We tested the monopeptide vaccine, thus establishing a proof of concept for the validity of the technique. We synthesized a peptide of 22 amino acids in length, corresponding to one of the candidate vaccines, and prepared a dendritic cell (DC) vaccine vector loaded with the 22 amino acids SARS-CoV-2 peptide (positions 50-71) contained in the NTD domain (DC-CoVPSA) of the Spike protein. Next, we tested the immunogenicity, the type of immune response elicited, and the cytokine profile induced by the vaccine, using a non-related bacterial peptide as negative control. Our results indicated that the CoVPSA peptide of the Spike protein elicits noticeable immunogenicity in vivo using a DC vaccine vector and remarkable cellular and humoral immune responses. This DC vaccine vector loaded with the NTD peptide of the Spike protein elicited a predominant Th1-Th17 cytokine profile, indicative of an effective anti-viral response. Finally, we performed a proof of concept experiment in humans that included the following groups: asymptomatic non-active COVID-19 patients, vaccinated volunteers, and control donors that tested negative for SARS-CoV-2. The positive control was the current receptor binding domain epitope of COVID-19 RNA-vaccines. We successfully developed a vaccine candidate technique involving optimizing lambda-superstrings and provided proof of concept in human subjects. We conclude that it is a valid method to decipher the best epitopes of the Spike protein of SARS-CoV-2 to prepare peptide-based vaccines for different vector platforms, including DC vaccines.

## Introduction

The coronavirus disease 2019 (COVID-19) epidemic represents the greatest global threat to human health at the current juncture, with more than 281 million people infected and more than 5.4 million mortalities worldwide since the disease was detected two years ago^1^. To end this epidemic, different types of vaccines are being developed in an accelerated manner^2–6^.

Although new cutting-edge technologies are being used in the production of vaccines, such as the development of mRNA vaccines^7, 8^, which speeds up the manufacturing process and reduces the cost of fabrication, they do not take into account the mutations that arise as the pandemic progresses.

Several effective vaccines against severe acute respiratory syndrome coronavirus 2 (SARS-CoV-2), the causative agent of COVID-19, are currently available, such as the Pfizer, Moderna, Oxford Vaccine Group/AstraZeneca, Janssen, BIOCAD, and CanSino Biologics vaccines. Clinical trials, as well as data from ∼50%–70% of vaccinated individuals in Europe and EEUU, show that the highest protection corresponds to the Pfizer (93%) and Moderna (90%) vaccines; the duration of this protection still requires evaluation. Thus, despite the important and promising advances that have been made in the design and development of vaccines against SARS-CoV-2, uncertainties still remain^9, 10^, making it imperative to continue the search for new candidate vaccines (CVs).

Several tools from computational biology are being used for different tasks in the fight against COVID-19, such as modeling^11^, identifying epitope maps^12^, designing protein inhibitors^13^, identifying inhibitors of the interaction of the Spike protein with angiotensin-converting enzyme 2 (ACE2) receptor^14^, optimizing antibodies^15^, and designing CVs^16–19^.

We present here two peptide-based CVs for SARS-CoV-2 designed entirely using computational methods and advanced tools of artificial intelligence. For one of these peptides, we provide proof of concept for immunogenicity.

Our technique is based in the concept of λ-superstring, introduced by our research group in a previous publication^20^. In that paper, we presented a new criterion for the selection of epitopes in the design of vaccines that was well suited to consider all the mutations, providing a balance with respect to the number of epitopes covered by the CV in the mutated versions of the target protein. Using this method, we consider the mutations in the pathogen’s genome and develop a CV that performs well against all those mutations.

Specifically, we considered a set of target strings, formed by the epitopes that can be selected for the CV, and a set of host strings, constituted by the different variants of the target protein, in which the known mutations are considered. In that context, given the value of a parameter λ, a λ-superstring is a sequence of amino acids with properties that ensure that the string covers at least λ epitopes in each of the host strings.

The concept of λ-superstring was generalized in our subsequent publication^21^ to that of a weighted λ-superstring by allowing the epitopes to be weighted by estimations of their immunogenicities. This generalization entails an important improvement in the applications to vaccine design^22^, as this consideration of an epitope’s immunogenicity more closely models biological and medical practice and increases the likelihood that the obtained CVs are effective. In fact, the use of weighted λ-superstrings could be useful in response to the high mutability of  viruses such as human immunodeficiency virus (HIV), hepatitis C virus (HCV), and influenza, and the generation of escape mutations.

Peptide-based vaccines against SARS-CoV-2 are developed using recombinant technology, which is the most widely used vaccine strategy. In fact, 50 protein or peptide constructs are in pre-clinical trials and 8 out of these 50 are in clinical trials, being the receptor binding domain (RBD) region of the Spike protein a common epitope of COVID-19 vaccines^3^, as well as an epitope detected in antibodies and T cells from COVID-19 patients^23, 24^. These studies also indicated that the RBD region was not the only B or T cell epitope detected in patients, with other parts of the Spike protein also being detected. Therefore, peptide-based vaccines for SARS-CoV-2 should include other epitopes of the Spike protein to maximize the broad spectrum of cellular immune responses they might elicit.

The success of peptide-based vaccines relies on three criteria: (1) easy to manufacture, (2) cost-effective production, and (3) high safety profile compared with whole virus vaccines^3, 23, 25^. However, such vaccines also have limitations, including the fact that they require specific methodologies to design epitopes with high immunogenicity and to test their efficacy as vaccines. To address this issue, we used two combined methods in the development of COVID-19 peptide-based vaccines. First, a computational technique that uses advanced tools of artificial intelligence to design the best immunogenic epitopes^20, 21^. Second, subcutaneous inoculation of DC vaccine platforms loaded with these peptides to test the delayed type hypersensitivity (DTH) reactions in mice, as an in vivo measurement of cellular immune responses that provides easy preparation for the clinic^22^.

To establish an experimental proof of concept of the method of using λ-superstrings, in this work we used Integer Programming to obtain the best solution for different lengths of the CV, to achieve the maximum value of λ, and therefore optimal immunological protection.

We used the Spike protein as the target, which is the best suited antigen for SARS-CoV-2 vaccine research, because in addition to being an intermediate in the interaction with host cell binding to the ACE2 receptor, it is also a surface-exposed protein^26, 27^, which makes it a suitable target protein for vaccine development.

In this way, we obtained CVs that offer potential protection against all the virus variants considered for the study (in this case, all the sequences appearing in the GenBank^28^ and GISAID^29^ websites until March 4, 2020). Our  objective was not only to develop an effective vaccine for the current pandemic, but also to confer protection against potential coronavirus mutations by considering sufficiently high values of λ. Thus, although future mutations might diminish the value of λ when new host strings associated to mutations appear, λ will nevertheless remain sufficiently high to be medically effective and inhibit the expansion and possible resurgence of the virus.

After obtaining the sequences, to select the most promising epitopes for the vaccine, we first associated a weight to each potential 9-mer epitope. More precisely, we used an aggregate function that combines estimations of the immunogenicity and the HLA-binding affinity of class I of these potential epitopes. The procedure is outlined briefly below, but is described in more detail in the “Methods” section. We used the “T cell class I pMHC immunogenicity predictor”^30^ of IEDB for the estimation of immunogenicity, and the “Peptide binding to MHC class I molecules”^31^ tool of IEDB for the estimation of HLA-I binding affinity. Since we had no specific information about the alleles of the different patients (whose number is much greater than the number of host strings) and our vaccine is not a personalized vaccine, we weighted each allele with its frequency appearing in “The Allele Frequency Net Database”^32^ that corresponds to the alleles of the HLA-I allele reference set previously published ^33^.

We chose these two variables, immunogenicity and HLA-I binding affinity, because of the key need to engage T cells in the development of an effective vaccine response against SARS-CoV-2 to ensure long-lasting immunity^34^.

We did not consider estimations of HLA-II binding affinity for three reasons. First, antigen presentation of viral proteins is mainly restricted by HLA-I molecules. Second, the computational complexity of the Integer Programming problem increases considerably, and it becomes impractical to obtain solutions in the range of lengths that we have considered. Moreover, it takes a prohibitive time for the algorithm to finish and requires a large amount of computer RAM. Third, prediction algorithms for MHC class II presentation are less accurate compared with class I algorithms^35^.

Besides, although HLA-II binding affinity was not considered in the optimization, the experimental results have shown a strong humoral immunity elicited by the CV, as is indicated in the “Methods” section.

To assure the antigenicity of our CVs we used the VaxiJen^36^ tool, which is a server for alignment-independent prediction of protective antigens that uses bacterial, viral and tumour protein datasets to derive models for prediction of whole protein antigenicity. We selected from among the 272 candidates those that exceeded the threshold to be considered probable antigens for the VaxiJen viral model.

As a result of our computations, we present in this work a map of optimal CVs with lengths varying from 9 to 280 amino acids, which on one hand optimize both immunogenicity and HLA-binding affinity, and on the other hand, confer balanced protection against all of the sequenced variants of COVID-19 surface protein obtained up to the moment at which the data were collected.

Then, to test the efficacy of our method, we first selected from this map a peptide of 22 amino acids contained within the NTD domain of the Spike protein (50–71 amino acids in the surface glycoprotein in the NCBI Reference Sequence YP_009724390.1). We selected a 22-mer of the NTD domain since it has a length feasible to be synthesized at high purity, it is an optimal length for loading onto DCs that admit epitopes lower than 30-mer^22^, and because a 22-mer of *Listeria monocytogenes* has previously shown high immunogenicity as CV, as well as effective protection^22^. Next, we incorporated the peptide into DC-based vaccine vectors to explore the epitope safety and immunogenicity, and to determine the type of peptide-induced immune response.

Finally, we performed a small proof of concept experiment in COVID-19-vaccinated volunteers and detected high levels of neutralizing IgG COVID-19 antibodies against the NTD domain that indicated a protective response.

## Methods

### Setting of the problem

Given two sets $H$ and $T$ of strings, called host strings and target strings, respectively, and given a mapping $w:T\to {\mathbb{R}}$ , we say that a string $s$ is a weighted λ–superstring^21^ if, for every $h\in H$ , the inequality $\sum_{t\in CS(h,s)\cap T}w(t)\ge \lambda$ holds, where $CS(h,s)$ is the set of common substrings of $h$ and $s$.

We solved in this work an instance of one of the combinatorial optimization problems settled in^21^: given a fixed length, find a λ-superstring with the maximum value of λ.

### Extraction of the sequences

The sequences were taken from two sources, the GenBank database^28^ and GISAID^29^ (using the sequences available up to March 4, 2020).

To search the GenBank database, the search term “Severe acute respiratory sindrome coronavuirus 2” AND “Homo sapiens” was applied for a nucleotide search. The results of the search were saved as Coding Sequences in the FASTA protein format to obtain information about the corresponding amino acids. Then, in the generated file,  sequences corresponding to “surface” or to “spike” protein (which both refer to  the same protein) were selected.

To search GISAID, the term “Human” was selected in the host window and “complete” (> 29000 bp) was also selected to obtain only complete genomes in human hosts. Information about the surface protein was extracted from the genomes using the GeneWise^37^ tool, by inserting the sequence of aminoacids corresponding to the surface glycoprotein (YP_009724390.1) product in the reference genome (NC_045512.2) into the protein window.

Duplicated sequences were removed, as well as sequences containing ambiguous characters such as “X”, “B”, “Z”, “J”, “O”, “U”, “.”, “*”. An anomalous short sequence of 35 amino acids was also discarded.

The resulting 22 sequences were taken as host strings, constituting the set $H$. The multiple alignment of the sequences, obtained using BioEdit, can be found in Additional file 1.

### Weighting of the epitopes

The set $T$ of target strings was taken to be the set of 9-tuples of elements of $A$ (where $A$ is the set of 20 amino acids) that are contained in at least one host string and that correspond to residues 1 to 1208, located before the transmembrane domain^38^.

The weight $w(s)$ associated to a target string (epitope) $s$ was calculated is as follows:

1. The estimation $i(s)$ of the immunogenicity of $s$ was calculated with the “T cell class I pMHC immunogenicity predictor” of IEDB.
2. The set $AI(s)$ of alleles of the HLA-I allele reference set with the “Peptide binding to MHC class I molecules” tool of IEDB which pass the threshold was computed, and the number $bI\left(s\right)=\sum_{i\in AI(s)}f(a)$ was calculated, where $f(a)$ is the estimated global frequency of allele $a$ in “The Allele Frequency Net Database”.

Next, the normalized families were computed as follows:

$${i}_{N}\left(s\right)=\frac{i\left(s\right)-{m}_{i}}{{M}_{i}-{m}_{i}},\,\,\mathrm{ where }\,\,{m}_{i}={min}_{s\in T} i\left(s\right)\,\,\mathrm{ and }\,\,{M}_{i}={max}_{s\in T} i(s),$$

$${bI}_{N}\left(s\right)=\frac{bI\left(s\right)-{m}_{bI}}{{M}_{bI}-{m}_{bI}}, \,\,\mathrm{where}\,\, {m}_{bI}={min}_{s\in T} bI\left(s\right)\,\, \mathrm{and}\,\, {M}_{bI}={max}_{s\in T} bI\left(s\right).$$

Finally, the weight of epitope $s$ was taken as

$$w\left(s\right)=\frac{3 {i}_{N}\left(s\right)+{bI}_{N}(s)}{4}.$$

The ponderation of the immunogenicity was taken to be larger than that of the binding affinity to favor the former, taking into account that it is a deterministic estimation, while the latter is a probabilistic estimation based on the frequencies of the most frequent alleles, and it does not cover exhaustively all possible alleles.

The target strings and weights can be found in Additional file 2.

### Optimization with CPLEX

CPLEX Optimizer^39^ was used with an Intel(R) Xeon(R) CPU E5-4620 v2 @ 2.60 GHz Processor with 512 GB of RAM to solve the Integer Programming algorithm described previously ^21^ to maximize the value of λ for a fixed length, with the set of host strings described in “Extraction of the sequences” and the set of target strings and weights described in “Weighting of the epitopes”.

The Integer Programming is founded in a graph theoretic formulation of the optimization problem based in a generalization of the Traveling Salesman Problem.

### Ranking the candidates with Vaxijen

The bioinformatics tool Vaxijen^36^ was used with each one of the candidates obtained in the optimization with CPLEX. “Virus” was selected as the target organism. The overall prediction for the protective antigen was calculated for each sequence and those over the threshold of 0.4 established for this model were selected, which is the threshold of the highest accuracy value beyond which the sequence is considered to be a probable antigen.

### Peptides

The sequence of different peptides from the Spike protein of SARS-CoV-2 were confirmed according to the published reference cryo-EM structure of the protein^38^. The peptide comprising amino acids 50–71 of the Spike protein with the sequence STQDLFLPFFSNVTWFHAIHVS, which is contained in the NTD domain (here designated as CoVPSA), as well as a non-related peptide of the same length selected from another pathogen unrelated to SARS-CoV-2, namely the Lmo 2459 virulence factor of *L. monocytogenes*, here referred as control peptide (CONT) with the sequence MTVKVGINGFGRIGRLAFRRIQ, or the  RBD peptide of SARS-CoV2 Spike protein S1 from amino acids 330–530, were all synthesized by Genescript with a purity ≥ 99% by HPLC.

### Preparation and safety of DC vaccines loaded with COVID-19 peptides

Bone-marrow-derived DCs were obtained from femurs of 8–12-week-old female mice and were cultured at 2 × 10^6^ cells/mL in six-well plates in Dulbecco’s Modified Eagle’s Medium (DMEM) supplemented with 20% fetal calf serum, 1 mM glutamine, 1 mM nonessential amino acids, 50 µg/mL gentamicin, 30 µg/mL vancomycin (DMEM complete medium), and 20 ng/mL granulocyte–macrophage colony-stimulating factor (GM-CSF). On Day 7, cells were harvested and analyzed by fluorescence-activated cell sorting (FACS) to evaluate cell surface markers. Differentiated DCs showed a phenotype of 98% CD11c^+^MHC-II^+^CD11b^-/+^CD40^-^CD86^-^ cells and were used in vivo for T cell responses.

The safety of DC vaccines loaded with peptides was explored with two assays to assess cell viability and apoptosis. Safety was considered optimal if the percentages of cell viability were higher than 95% and apoptosis induction was no higher than 6%–7%. Cell viability was explored after DC incubation with synthesized peptides at a concentration of 50 µg/mL for 16 hours, cells were then washed and stained with trypan blue. Results were expressed as the percentage viability compared with non-treated DCs of triplicate experiments ± SD (P < 0.05). Apoptosis was measured after labeling with the DNA fluorescent intercalating probe 7-aminoactinomycin D (7-AAD, BD Biosciences, San Jose, CA, USA) and cell surface analysis of the apoptotic marker, Annexin V conjugated with allophycocyanin (APC) fluorochrome, followed by incubation of DCs with peptides for 16 hours. Staining of DCs with 7-AAD corresponded to normal cell death and staining of DCs with annexin-V alone indicated the percentage of apoptotic cell death. The results were expressed as the mean ± SD (P < 0.05).

### T cell responses elicited by DC-vaccines loaded with COVID-19 peptides

For DTH analysis, C57BL/6 mice that had been primed for 7 days intraperitoneally with COVID-19 peptides (50 µg/ml) were inoculated into the left hind footpad with DC vaccines (10^6^ cells/mouse) pre-loaded with COVID-19 peptide or a control peptide protective against another unrelated infection (a 22-mer of the glyceraldehyde-3-phosphate-dehydrogenase of *L. monocytogenes*)^22^. DC vaccines were formulated in the presence of DIO-1 (2 μg/mL)^22^. Negative controls were the right hind footpads, since they were not inoculated. After 48 hours, the footpad thickness was measured with a caliper and the results were expressed in millimeters as the mean of three different experiments. Next, the popliteal lymph nodes were collected and homogenized, and cell homogenates were passed through cell strainers to analyze CD4^+^ and CD8^+^ T cells by flow cytometry. The results are expressed as the percentage of positive cells ± SD.

### Cytokine measurements

Cytokines in mice sera, DCs, or mouse DC supernatants were quantified using multiparametric Luminex kits. In brief, interferon gamma (IFN-γ), IL-2, IL-4, IL-6, IL-10, IL-12 (p70), IL-17A, KC/CXCL1, MIP-2, and TNF-α levels in mice serum samples were quantified using the Luminex 200 platform with a magnetic system (Milliplex MAP Mouse High Sensitivity T Cell Magnetic Bead Panel, EMD Millipore Corporation, Billerica, MA, USA) following the manufacturer's instructions. Cytokine concentrations are expressed as the average of three replicates in pg/mL ± SD. Similarly, cytokines in the human sera of COVID-19 patients, and vaccinated or control volunteers were quantified using the multiparametric Luminex kit (Milliplex human 1xl HSTCMAG-28SK including the following cytokines: IFN-γ, IL-10, IL-17A, IL-2, IL-4, IL-6, IL-8, TNF-α, EMD Millipore Corporation) following the manufacturer´s instructions.

### ELISA measurements of antibodies

Neutralization COVID-19 IgG antibodies were measured with the cPass™ kit from GenScript supplied by IES Medical (Leioa, Bizkaia, https://www.iesmedical.es) that measures the percentage of antibodies able to neutralize the SARS-CoV-2 virus in human sera. Antibodies of IgG isotype against the peptides RBD, CoVPSA, or CONT were assessed according to previously reported methods^40^. In brief, ninety-six well plates were coated with the different peptides (RBD and CoVPSA from the Spike protein and CONT from the Lmo2459 virulence factor) at a concentration of 50 µg/mL in carbonate buffer (pH 8.0) at 4ºC overnight, and were then washed and incubated with 1 mg/mL of BSA (fraction V)  for blocking non-specific sites. The sera of COVID-19 patients with non-active infection (four patients: COV-1: NAT, COV-2: MAR, COV-3: AMA, COV-4: MABR), vaccinated volunteers with different COVID-19 vaccines (three with Pfizer and one with Moderna) (four volunteers: VAC-1: HEC, VAC-2: DAV, VAC-3: CAR, VAC-4: EDU), and healthy donors that were non-infected, non-vaccinated, and tested negative in a COVID-19 antigen test (four volunteers: CONT-1: CAD, CONT-2: AND, CONT-3: VIC, CONT-4: EFG) were 1/10 diluted and peptide-coated plates were incubated with the diluted sera for two hours at room temperature. Reactions were developed with goat anti-human IgG and the absorbances analyzed at 450 nm and expressed as optical units (OD) from the mean values ± SD of triplicate experiments (*P* < 0.05). Only results with OD ≥ 0.2 ± 0.01 were considered positive in Fig. 4.

### Statistical analysis

For statistical analysis, the Student´s *t*-test was applied to all mouse assays and ELISA assays. All samples were evaluated in triplicate and experiments were performed at least three times. GraphPad software was used for the generation of all graphs presented.

### Ethics statement

This study was carried out in accordance with the Guide for the Care and Use of Laboratory Animals of the Spanish Ministry of Science, Research and Innovation. The Committee on the Ethics of Animal Experiments of the University of Cantabria approved the protocol (Permit number: PI-10-17) that follows the Spanish legislation (RD 1201/2005). All surgeries were performed by cervical dislocation, and all efforts were made to minimize animal suffering. For the human data from COVID-19 patients and vaccinated non-infected donors, the study was approved by the Ethical Committee of Clinical Research of Cantabria at Instituto de Investigación Marqués de Valdecilla (Santander, Spain), reference number Acta 13/2021. All participants signed the Informed Consent documents and received and Information Document about the project. These documents are in the custody of physicians in accordance with Spanish Law (Ministry of Health). Similarly, for the use of human data on sera from COVID-19 patients, vaccinated volunteers, and healthy donors, all participants signed an Informed Consent form and received a General Project Information document, following approval from the Committee of Clinical Ethics of Cantabria (CEm) entitled: Trained immunity in the design of COVID-19 nanovaccines (reference INNVAL20-01).

## Results

As described in the “Methods” section, the problem of finding optimal weighted λ–superstrings with a maximum value of λ for a given length that can serve as CVs against SARS-CoV-2 was solved using an Integer Programming algorithm (Methods, “Optimization with CPLEX”). This algorithm was fed with three elements, the host string set $H$, the target string set $T$ and the weighting function $w$, obtained as follows:

First, the set $H$ of host strings was taken as the 22 distinct sequences corresponding to the Surface protein of SARS-COV-2 that appear in the Genbank^28^ and GISAID^29^ databases (Methods, “Extraction of the sequences”).

Next, we took as the set $T$ of target strings (i.e., as potential epitopes), the 9-mers contained in some of the 22 host strings in positions corresponding to residues before the transmembrane domain.

Then, we assessed the weights of the epitopes using a function $w$ in which the estimation of their immunogenicities and the estimation of the binding affinity to HLA-I was taken into account (Methods, “Weighting of the epitopes”).

**Figure 1:**
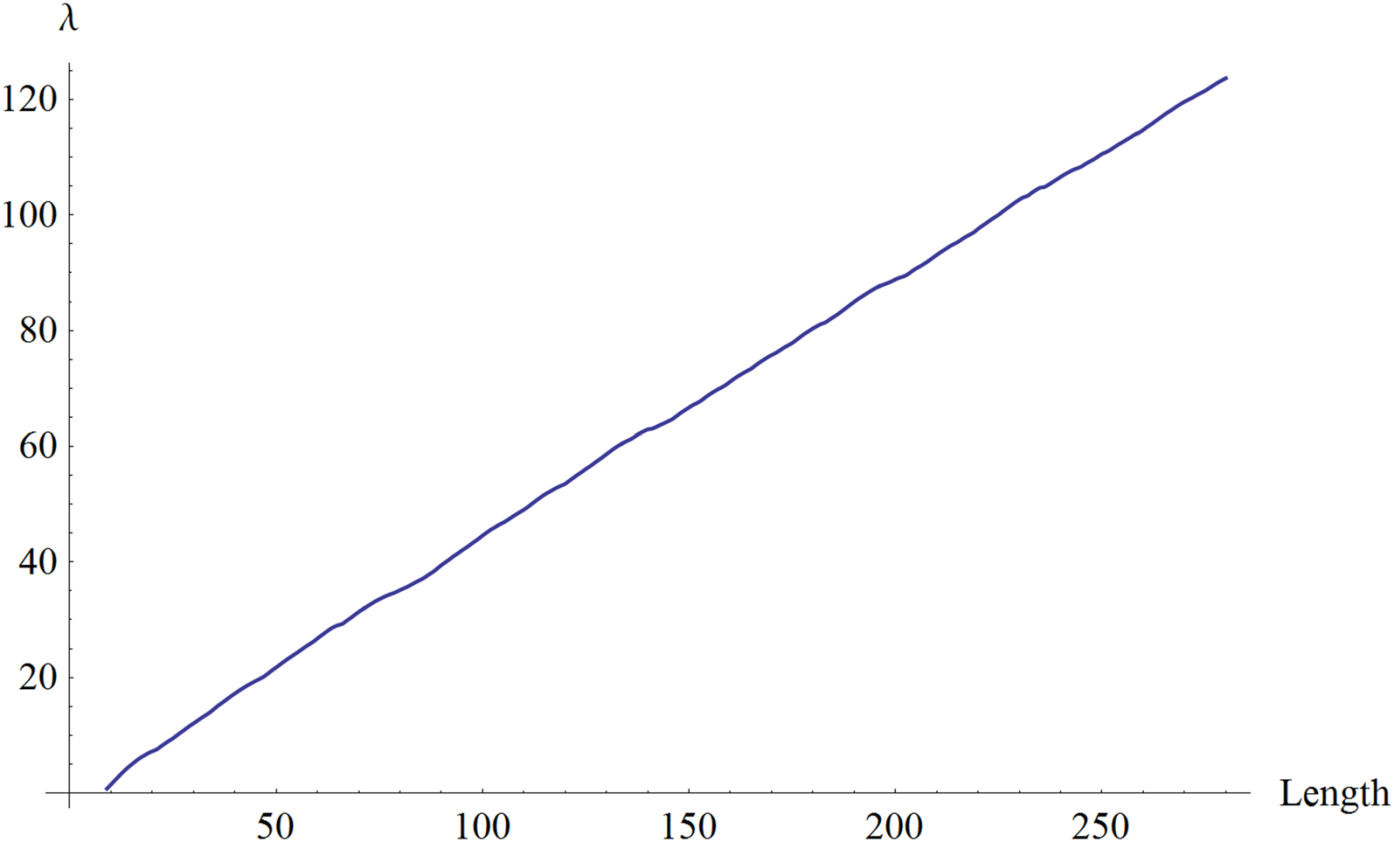
Scatterplot for λ. The abscissa axis shows the length of the candidate peptides, the ordinate axis shows the value of λ.

Then, we used the algorithm to calculate a weighted λ–superstring with maximum λ for each length between 9 and 280. A scatterplot for the value of λ as a function of the length of the CV is shown in Fig. 1. It can be well fitted by a least square line with the regression line $\uplambda =-0.579005+0.446982\cdot l$ , where $l$ is the length of the candidate. The intercept and slope of the line were accurately determined, with a low standard error and a low P-value, as shown in Table 1. The R-squared value of the fit was 0.999668, and the closeness of this value to 1 indicates a good fit. Thus, each one-unit increase in length is associated approximately with an increase of 0.4 in λ all along the range from 9 to 280. Therefore, the map is robust and there is no significant loss in the λ increase per unit length in the considered interval of lengths.

**Table 1:** Inference for the intercept and slope constants.

	Estimate	Standard error	P-value
$1$	−0.579005	$0.0815211$	$1.08742\times {10}^{-11}$
$x$	0.446982	$0.000495704$	$1.07761\times {10}^{-471}$

Furthermore, we calculated the VaxiJen overall prediction for each CV (Methods, “Ranking the candidates with Vaxijen”). These optimal weighted λ-superstring, as well as the corresponding λ values and VaxiJen predictions, are shown in the table in Additional file 3. The threshold of 0.4 indicated in VaxiJen for the viral model was surpassed by the candidates with lengths of 22,24,67,68,69,70, and 175, as well as those with a length of at least 184 amino acids (candidates shown in green in the above-mentioned table).

Each λ-superstring can be naturally divided as it constitutes a union of a small number of peptides located in different regions of the protein. These peptides are enumerated, for each λ-superstring, in the fourth column of the table. When a peptide has some intersection with a domain of the protein, the domain is annotated next to the peptide. For the λ-superstrings with lengths from 176 to 183, the third peptide intersects two domains, namely NTD and RBD, and for those with lengths from 237 to 247, the fourth peptide also intersects the same two domains.

The two CVs with the maximum value in the VaxiJen overall predictions are shown in Table 2. The first CV (22 amino acids in length) is contained in the NTD domain, and the second CV (277 amino acids in length) can be divided, as previously described, as it originates a multipeptide of five peptides. In particular, the third and fifth peptides intersect the NTD and RBD domains, respectively, making them appropriate targets for vaccine development against SARS-CoV-2^27^.

**Table 2:** Optimal weighted λ–superstring, λ values, and VaxiJen overall prediction for the two candidate vaccines with the maximum λ value.

Length	Lambda	Prediction	Sequence
22	8.03	0.5545	1:STQDLFLPFFSNVTWFHAIHVS(NTD)
277	122.44	0.5190	1:QSAPHGVVFLHVTYVPAQEKNFTTAPAICHDGKAHFPREGVFVSNGTHWFVTQRNFYEPQIITTDNTFVSGNC(CD) 2:TEVPVAIHADQLTPTWRVYSTGSNVFQTRAGCLIGAEHVNNSYECDIPIGAGICASYQTQ 3:DLFLPFFSNVTWFHAIHVSGTNGTKRFDNPVLPFNDGVYFASTEKSNIIRGWIFGTTLDSK(NTD) 4:FLPFQQFGRDIADTTDAVRDPQTLEILDITPCSFGGVSVITPGTNTSN 5:FRVQPTESIVRFPNITNLCPFGEVFNATRFASVYA(RBD)

Column 1, the number of amino acids in the CVs; column 2, the value of λ; column 3, the VaxiJen overall prediction for antigenicity; column 4, the peptides whose union forms the λ-superstring.

We selected the first CV in Table 2 for further biological assays because it showed the maximum value in the overall prediction in VaxiJen. This peptide STQDLFLPFFSNVTWFHAIHVS is 22 amino acids in length, and is contained in the NTD domain, therefore being a valid candidate antigen for vaccine development^27, 41, 42^, with an overall prediction of 0.5545 in VaxiJen.

After analyzing our results computationally, we synthesized the 22-amino acid SARS-CoV-2-NTD peptide (designated here as CoVPSA) and performed in vivo experiments to test its immunogenicity and putative efficiency.

A first proof of concept was related with the immunogenicity of CoVPSA, and it was determined using a previously described procedure^22^ that evaluates the best immunogenic epitopes for preparing vaccines.

Safety was also examined by a cell viability assay after Trypan blue staining and apoptosis induction. Safety for DC vaccine vectors is considered as a percentage of cell viability higher than 95% and apoptosis induction lower than 7%–8%. Table 3 shows that DC vaccines loaded with CoVPSA peptides, or the unrelated bacterial peptide used as negative control, presented 98%–99% cell viability and lower than 4%–5% apoptosis. Therefore, we concluded that the DC vaccines loaded with peptides presented good safety profiles.

**Table 3:** Safety of DC vaccines loaded with COVID-19 peptides.

Condition^a^	Cell viability^b^	Apoptosis^c^
NT	99 ± 0.3%	3 ± 0.3%
CoVPSA	98 ± 0.2%	4 ± 0.2%
DC-peptide CONT	97 ± 0.5%	4.5 ± 0.4%

^a^DC cells were incubated with 50 µg/mL of peptides for 16 hours.

^b^Cell viability was explored after trypan blue staining and microscopy was used to count viable (non-stained) and non-viable (blue stained) cells. Results are expressed as the percentage of viable versus total cells (viable and non-viable cells).

^c^Apoptosis is detected after DC staining with the DNA marker 7-AAD-PE and the apoptotic marker Annexin-V-APC. Results show the percentage of apoptotic cells ± SD of triplicate samples. All experiments were performed at least three times.

Next, we performed immunogenicity assays to measuring the DTH response of the vaccine vector. DCs were loaded with the peptide, then, mice were primed for 7 days intraperitoneally with COVID-19 peptides and then inoculated with the vaccine formulation (DC-CoVPSA) into the left hind footpads, with the non-inoculated right hind footpads acting as basal controls. Forty-eight hours later, we measured the DTH response as the swelling of the left hind footpads compared with the right hind footpads.

**Figure 2:**
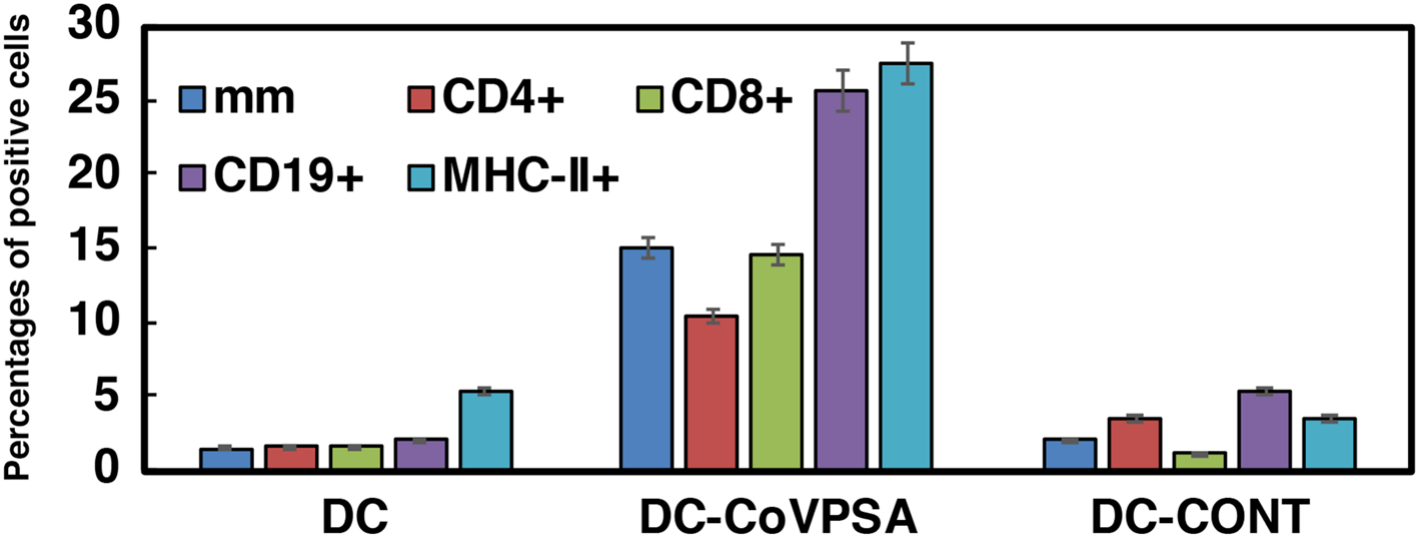
Immunogenicity of the CoVPSA peptide in vaccine platforms. The hind footpads of mice (C57BL/6, n = 5) were inoculated with the DC vaccine (10^6^ cells/mouse) loaded with different peptides (COVID-CoVPSA or peptide control, CONT) or remained as empty DCs in formulations with the adjuvant DIO-1 (40 ng/mL). After 48 hours, footpad swelling was measured with a caliper (dark blue bars) and expressed as the difference in mm between left and right hind footpads. Results are the mean ± SD of three different experiments (*P < 0.05). Popliteal lymph nodes were then isolated from the legs of the mice and after homogenization, immune cell populations were analyzed by flow cytometry. The percentages of CD4^+^ (red bars), CD8^+^ T cells (green bars), CD19 + (B cells, purple bars), and MHC-II + positive cells, mainly DCs or macrophages (light blue bars), are shown. Results are expressed as the percentage of positive cells ± SD of three different experiments (*P* < 0.05). Experiments were performed six times.

**Figure 3:**
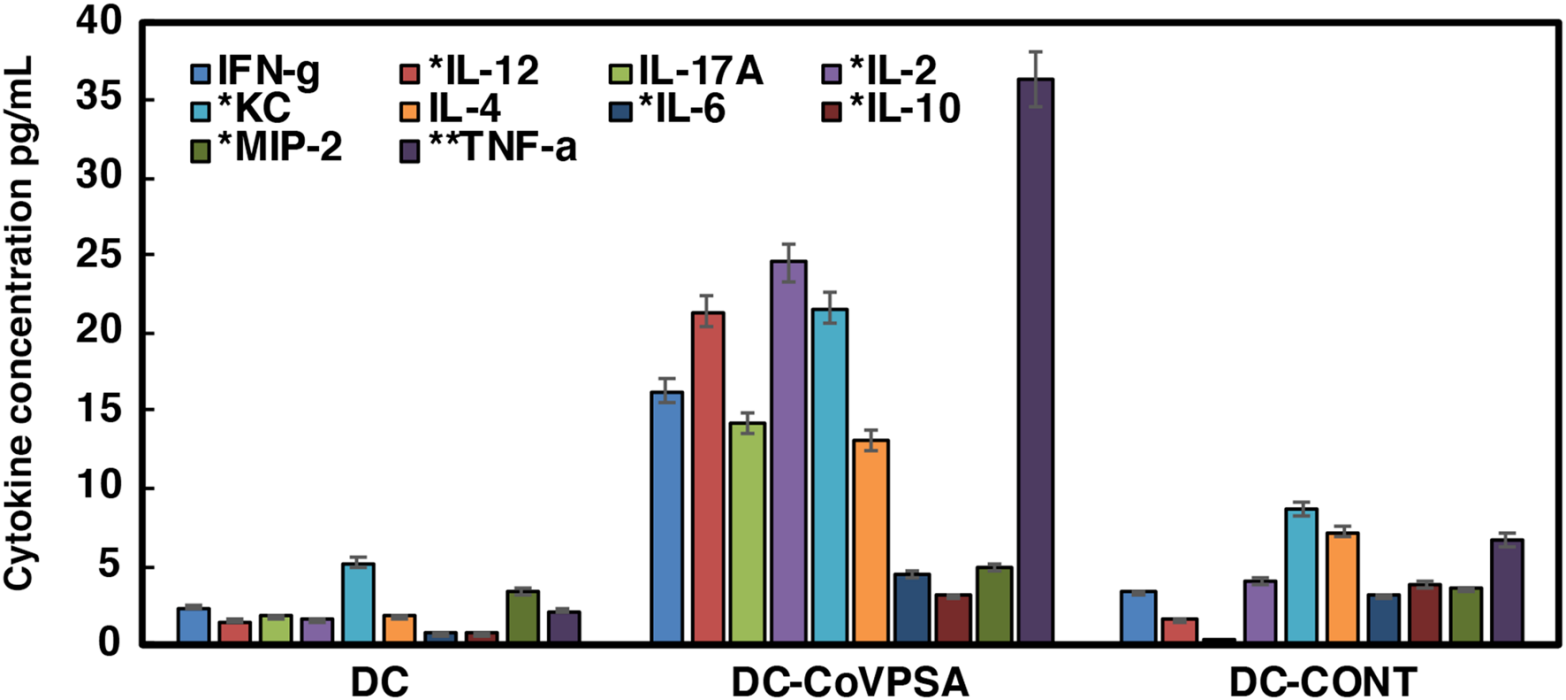
Cytokine levels of mice inoculated with DC vaccine platforms. Cytokine levels were detected in the sera of mice, as described in Fig. 2 , and were measured using a multiparametric Luminex kit from Merck. Results are expressed as pg/mL of each cytokine ± SD of triplicate samples (*P* ≤ 0.05). Asterisk: Levels of cytokines should be multiplied tenfold. Double asterisk: Levels of cytokines should be divided twofold. Cytokine experiments were performed five times.

We also included empty DCs in these experiments and DCs loaded with a bacterial peptide unrelated to SARS-CoV-2 but with high CV efficiency against the bacterial pathogen^22^. Analysis of DTH responses (blue bars in Fig. 2) indicated that DCs loaded with COVID-19 designed peptide (DC-CoVPSA bars) elicited significantly stronger immune responses than DCs loaded with the control bacterial peptide (DC-CONT bars) or empty DCs (DC labelled bars). This may be explained by the fact that mice primed and DC-vaccinated with the same COVID-19 peptide produced high DTH responses, while mice primed and DC-vaccinated with different peptides were not able to elicit significant DTH responses (DC-control in Fig. 3). Next, we collected the popliteal lymph nodes and cultured them in vitro with 1 µg/mL of each peptide, CoVPSA, control peptide, or saline for 72 hours to examine the main immune cell populations by flow cytometry.

We observed that the highest percentages of immune cells corresponded to CD19^+^ cells (25,63%) that usually correspond to B cells, followed by MHC-II^+^ cells (27, 45%) that usually label DCs and macrophages, next CD4^+^ T cells (10,39%) and CD8^+^ T cells (14,61%).

The control peptide (CONT) produced no significant immune responses, as we observed only a small percentage of CD19^+^ cells (5,3%) and moderate numbers of MHC-II^+^ cells (13,5%) (DC-CONT bars in Fig. 2). Empty DCs (DC bars in Fig. 2) induced no significant numbers of immune cells.

These results indicated the clear induction of immune cells by DC vaccines loaded with CoVPSA peptide, with immune cells involved in antibody formation, such as B cells, DCs, and CD4^+^ T cells, being stimulated. While not predominant, cytotoxic immune responses caused by CD8^+^ T cells were also induced by DC vaccines loaded with CoVPSA peptide. These results were not surprising since CD4^+^ and CD8^+^ T cell epitopes are recovered from patients with mild and severe COVID-19 that are specific for the Spike protein^24^.

A second proof of concept was related with the production of cytokines, either anti-viral cytokines, such as TNF-α, IFN-γ, IL-2, KC, and IL-12, or acute Th2 cytokines, such as IL-4, IL-6, MIP-2, or IL-10. The COVID-19 cytokine storm observed in patients with severe disease correlates with high levels of TNF-α, IL-6, IL-4, and IL-10, as well as with a clear deficiency in the production of IFN-related cytokines (i.e., IFN-α, IFN-γ, or IL-12)^43^.

Our results in Fig. 3 show that DCs loaded with CoVPSA peptide produced mainly Th1-Th7 cytokines, IL-12, IL-17A, and IL-2. However, this DC-CoVPSA vaccine platform did not induce cytokines participating in the COVID-19 cytokine storm, such as IL-6, IL-10, or TNF-α (bars labelled with DC-CoVPSA in Fig. 3).

Interestingly, DC-CoVPSA vaccines induced high levels of IFN-γ (blue bars in Fig. 3) but barely detectable levels of MIP-2, an inflammatory cytokine that recruits inflammatory macrophages (grey bars in Fig. 3). The lack of significant levels of IL-4 (orange bars in Fig. 3) but high levels of IL-2 (red bars) strongly suggested the induction of Th1-Th17-type immune responses, but with no exacerbation of other cytokines, such as TFN-α or MIP-2.

**Figure 4:**
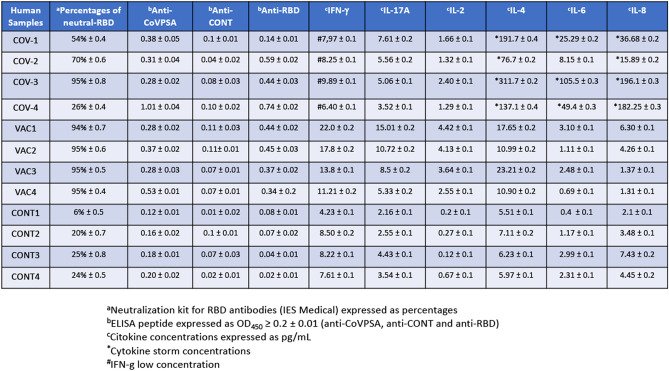
Correlation between neutralization IgG antibodies, anti-CoVPSA antibodies, and Th1-Th17 cytokines in COVID-19-vaccinated volunteers. The sera of 12 volunteers (four asymptomatic non-active COVID-19 patients: COV-1, COV-2, COV-3, and COV-4; four vaccinated volunteers who tested negative for SARS-CoV-2: VAC-1, VAC-2, and VAC-3 who received the Pfizer vaccine, and VAC-4 who received the Moderna vaccine; four control donors who were unvaccinated and tested negative for SARS-CoV-2: CONT1, CONT2, CONT3, and CONT4) were tested for IgG neutralization antibodies (IES Medical kit, first column), IgG anti-CoVPSA antibodies (second column), and IgG anti-RBD antibodies (third column). Reactions were developed with goat anti-human IgG and the absorbances (OD) were analyzed at 450 nm. Results are expressed as percentages of neutralization ± SD (^a^column), or as the mean ± SD of triplicate data.

In summary, the high levels of IFN-γ and especially IL-12, involved in vaccine efficiency and anti-viral responses, respectively, prompted us to suggest that CoVPSA peptide might function as an immunogenic epitope. CoVPSA peptide might be a good candidate to prepare vaccine platforms that induce not only antibody production but strong anti-viral T cell responses. We also confirmed these results in samples of human sera that served as a third proof of concept of our vaccine design. We recruited four asymptomatic patients with non-active COVID-19, four vaccinated volunteers (three with the Pfizer vaccine and one with the Moderna vaccine prepared against the RBD region of Spike protein), and four healthy donors that were non-vaccinated and tested negative in a COVID-19 antigen test. We collected blood from these 12 volunteers and compared the titers of different IgG COVID-19 antibodies as follows: (i) IgG antibodies able to neutralize the virus (neutral-RBD column in Fig. 4) were assessed by a neutralization antibody assay, (ii) anti-RBD antibodies that correspond to IgG antibodies recognizing the whole Spike protein including the RBD region binding to the ACE2 receptor (anti-RBD column), and (iii) anti-CoVPSA antibodies that reflect the IgG antibodies against our designed peptide in the NTD region (anti-CoVPSA column). As expected, COVID-19 asymptomatic patients presented medium and varied titers of IgG viral neutralization antibodies and low but significant levels of whole Spike protein anti-RBD antibodies, as previously reported^44–46^. These COVID-19 patients also presented medium anti-CoVPSA IgG antibody titers (compare columns 1, 2 and 4 of Fig. 4). Interestingly, volunteers vaccinated with mRNA vaccines prepared against the RBD region of the Spike protein presented not only the highest titers of IgG viral neutralization antibodies that correlated with significant antibody titers against the RBD region^44–46^, but also significant responses against the CoVPSA peptide. Analyses of the cytokine concentrations in the sera of these volunteers indicated that COVID-19 patients presented a storm cytokine pattern with high levels of IL-4, IL-6 and IL-8 and low or insignificant levels of IFN-γ, as previously published^44–50^. Interestingly, vaccinated volunteers presented high levels of IFN-γ as well as significant levels of IL-2 and IL-4. This indicates that mRNA vaccines induce good antibody responses, as well as significant anti-viral cellular responses, as measured with neutralizing anti-RBD antibodies, antibodies against other Spike protein regions, such as the NTD region, and high levels of anti-viral cytokines, such as IFN-γ, IL-17A and IL-2.

## Discussion

In this work, we established a proof of concept for our computational vaccine design method using λ-superstrings, and we demonstrated the feasibility of this method to obtain effective CVs.

Unlike previous studies in the medical literature, we did not start from a single genome in our analysis, instead we considered several genomes corresponding to different mutated versions. Furthermore, unlike most of the vaccines currently developed, our candidate is a peptide vaccine that does not consider the entire Spike protein, but rather a set of computationally selected overlapping epitopes.

In our study, we first found a map of CVs against SARS-CoV-2 targeted to the Spike protein, in which the length of the candidates ranged from 9 to 280 amino acids. Then, we filtered the candidates of this map to those considered protective antigens according to the VaxiJen bioinformatics tool, and finally synthesized the candidate peptide with the highest value from the overall prediction. This peptide was 22 amino acids in length and comprised the sequence STQDLFLPFFSNVTWFHAIHVS, which is contained in the NTD domain of the protein.

To experimentally validate the viability of the candidate, we performed several in vivo assays. The result of these experiments was positive for the following reasons:

1. The selected CV elicited a robust immune response in mice with Th1-Th17 pro-inflammatory features and strong stimulation of cells involved in antibody production.
2. The selected CV elicited the production of anti-viral cytokines. The cytokine profile obtained was adequate because it mainly involved anti-viral cytokines IL-12, IL-17A, and IL-2, not cytokines participating in the COVID-19 cytokine storm, such as IL-6 or IL-10.
3. The DC vaccines loaded with peptides showed  good safety profiles.
4. mRNA vaccines recognizing the RBD region of Spike protein induce a wide anti-Spike protein immune response, since we also observed significant levels of antibodies against other parts of the Spike protein, such as the NTD region (the CoVPSA peptide).
5. Antibodies against the selected CV peptide, CoVPSA, were detected at good titers in COVID-19 patients, although the profiles varied (COV-4 patient was a good responder, whereas COV-1, COV-2 and COV-3 patients were medium responders, column 2 in Fig. 4). Interestingly, the vaccinated group that was inoculated with the RBD antigen (RNA against the SARS-CoV-2 RBD region) and not with the CoVPSA antigen, also presented significant levels of antibodies to anti-CoVPSA antigen (VAC-4 volunteer was a good responder, whereas VAC-1, VAC-2 and VAC-3 volunteers were medium responders, column 2 in Fig. 4). This supported the conclusion that CoVPSA might be a relevant antigen to incorporate in COVID-19 vaccines to induce high levels of anti-viral cytokines, such as IFN-γ, IL-17A, and IL-2.
6. COVID-19-vaccinated volunteers presented high titers of anti-COVID-19 neutralization antibodies, varied responses to all regions of the Spike protein (RBD or NTD (CoVPSA peptide) regions), and anti-viral Th1-Th17 cytokines. These three features are characteristic of efficient vaccines.

Taken together, these results confirm the use of λ-superstrings as an effective means of detecting feasible vaccines against SARS-CoV-2.

It is worth noting that we also showed that peptides targeting the NTD region of the Spike protein, which were already known to be a good target for antibodies^48–50^, can also induce potent cellular immunity. However, our techniques selected specific parts of the NTD region based on our combinatorial optimization method that considers all of the generated variants at once, and not the complete NTD region. Moreover, the selection of this area was not premeditated, but the respective weights of the peptides and the consideration of the whole virus variant directed the algorithm to choose epitopes in that region.

In future studies, it would be worthwhile testing the second CV in Table 2, which is a multipeptide, and also the other CVs indicated in green in Additional file 3, which are those that pass the threshold established in VaxiJen to be considered as probable antigens. This would increase the range of potentially effective vaccines against SARS-CoV-2 that would cover a high percentage of the population. The uncertainty surrounding the future effectiveness of currently available vaccines means that such endeavors may prove valuable.

In summary, we have proven that our methodology for designing CVs that utilizes λ-superstrings represents an efficient alternative approach to peptide-based vaccine design for SARS-CoV-2. This may aid the design of further safe and efficient COVID-19 vaccines in the near future.

## Supplementary Information

Supplementary Information 1.

Supplementary Information 2.

Supplementary Information 3.

## Data Availability

The datasets supporting the conclusions of this article are included within the article and its additional files.

## Abbreviations

ACE2: Angiotensin-converting enzyme 2 receptor
APC: Allophycocyanin
CV: Candidate vaccine
DC: Dendritic cell
DMEM: Dulbecco’s Modified Eagle’s Medium
DTH: Delayed type hypersensitivity
GM-CSF: Granulocyte-macrophage colony-stimulating factor
RBD: Receptor binding domain
7-AAD: 7-Aminoactinomycin D
