# Supplementary material for: First computational design using lambda-superstrings and in vivo validation of SARS-CoV-2 vaccine
Source: Sci Rep. 2022 Apr 19;12:6410. doi: 10.1038/s41598-022-09615-w (PMC9016385; doi:10.1038/s41598-022-09615-w)
Supplement: Supplementary file 1 — Supplementary Information 1. [file 41598_2022_9615_MOESM1_ESM.rtf]

Alignment of the 22 host strings

Multiple alignment of the sequences obtained with BioEdit software

              10        20        30        40        50        60           
     ....|....|....|....|....|....|....|....|....|....|....|....|
S1   MFVFLVLLPLVSSQCVNLTTRTQLPPAYTNSFTRGVYYPDKVFRSSVLHSTQDLFLPFFS 
S2   ............................................................ 
S3   ...........................N................................ 
S4   ............................................................ 
S5   ................................................Y........... 
S6   ............................................................ 
S7   ...............................I............................ 
S8   ............................................................ 
S9   ............................................................ 
S10  ............................................................ 
S11  ............................................................ 
S12  ............................................................ 
S13  ............................................................ 
S14  ----------------------------S............................... 
S15  ...............................V............................ 
S16  ............................................................ 
S17  ............................................................ 
S18  ............................................................ 
S19  ............................................................ 
S20  ............................................................ 
S21  ............................................................ 
S22  ............................................................ 

              70        80        90       100       110       120        
     ....|....|....|....|....|....|....|....|....|....|....|....|
S1   NVTWFHAIHVSGTNGTKRFDNPVLPFNDGVYFASTEKSNIIRGWIFGTTLDSKTQSLLIV 
S2   ............................................................ 
S3   ............................................................ 
S4   ............................................................ 
S5   ............................................................ 
S6   ............................................................ 
S7   ............................................................ 
S8   ............................................................ 
S9   ............................................................ 
S10  ............................................................ 
S11  ............................................................ 
S12  ............................................................ 
S13  ............................................................ 
S14  ............................................................ 
S15  ............................................................ 
S16  ............................................................ 
S17  ............................................................ 
S18  ............................................................ 
S19  ............................................................ 
S20  ............................................................ 
S21  ............................................................ 
S22  ............................................................ 

             130       140       150       160       170       180     
     ....|....|....|....|....|....|....|....|....|....|....|....|
S1   NNATNVVIKVCEFQFCNDPFLGVYYHKNNKSWMESEFRVYSSANNCTFEYVSQPFLMDLE 
S2   ............................................................ 
S3   ............................................................ 
S4   ............................................................ 
S5   ............................................................ 
S6   ............................................................ 
S7   ............................................................ 
S8   ............................................................ 
S9   ............................................................ 
S10  ............................................................ 
S11  ............................................................ 
S12  ............................................................ 
S13  ............................................................ 
S14  ............................................................ 
S15  ............................................................ 
S16  ............................................................ 
S17  ..................................................---------- 
S18  ............................................................ 
S19  ............................................................ 
S20  ............................................................ 
S21  ............................................................ 
S22  ............................................................ 

             190       200       210       220       230       240     
     ....|....|....|....|....|....|....|....|....|....|....|....|
S1   GKQGNFKNLREFVFKNIDGYFKIYSKHTPINLVRDLPQGFSALEPLVDLPIGINITRFQT 
S2   ............................................................ 
S3   ............................................................ 
S4   ........................................W................... 
S5   ............................................................ 
S6   ............................................................ 
S7   ............................................................ 
S8   ............................................................ 
S9   ............................................................ 
S10  ............................................................ 
S11  ............................................................ 
S12  ............................................................ 
S13  ............................................................ 
S14  ............................................................ 
S15  ............................................................ 
S16  ............................................................ 
S17  --------------------------.................................. 
S18  ............................................................ 
S19  ............................................................ 
S20  .....................N...................................... 
S21  ............................................................ 
S22  ............................................................ 

             250       260       270       280       290       300     
     ....|....|....|....|....|....|....|....|....|....|....|....|
S1   LLALHRSYLTPGDSSSGWTAGAAAYYVGYLQPRTFLLKYNENGTITDAVDCALDPLSETK 
S2   ............................................................ 
S3   ............................................................ 
S4   ............................................................ 
S5   ............................................................ 
S6   ......R..................................................... 
S7   ............................................................ 
S8   ............................................................ 
S9   ............................................................ 
S10  ............................................................ 
S11  ............................................................ 
S12  ............................................................ 
S13  ............................................................ 
S14  ............................................................ 
S15  ............................................................ 
S16  ............................................................ 
S17  ........S................................................... 
S18  ............................................................ 
S19  ............................................................ 
S20  ............................................................ 
S21  ............................................................ 
S22  ............................................................ 

             310       320       330       340       350       360     
     ....|....|....|....|....|....|....|....|....|....|....|....|
S1   CTLKSFTVEKGIYQTSNFRVQPTESIVRFPNITNLCPFGEVFNATRFASVYAWNRKRISN 
S2   ............................................................ 
S3   ............................................................ 
S4   ............................................................ 
S5   ............................................................ 
S6   ............................................................ 
S7   ............................................................ 
S8   ............................................................ 
S9   .....................................................D...... 
S10  ............................................................ 
S11  ............................................................ 
S12  ............................................................ 
S13  ............................................................ 
S14  ............................................................ 
S15  ............................................................ 
S16  ............................................................ 
S17  ............................................................ 
S18  ............................................................ 
S19  ............................................................ 
S20  ............................................................ 
S21  ............................................................ 
S22  ............................................................ 

             370       380       390       400       410       420     
     ....|....|....|....|....|....|....|....|....|....|....|....|
S1   CVADYSVLYNSASFSTFKCYGVSPTKLNDLCFTNVYADSFVIRGDEVRQIAPGQTGKIAD 
S2   ............................................................ 
S3   ............................................................ 
S4   ............................................................ 
S5   ............................................................ 
S6   ............................................................ 
S7   ............................................................ 
S8   ............................................................ 
S9   ...Y........................................................ 
S10  ......F..................................................... 
S11  ............................................................ 
S12  ............................................................ 
S13  ............................................................ 
S14  ............................................................ 
S15  ............................................................ 
S16  ............................................................ 
S17  ............................................................ 
S18  ............................................................ 
S19  ............................................................ 
S20  ............................................................ 
S21  ............................................................ 
S22  ............................................................ 

             430       440       450       460       470       480     
     ....|....|....|....|....|....|....|....|....|....|....|....|
S1   YNYKLPDDFTGCVIAWNSNNLDSKVGGNYNYLYRLFRKSNLKPFERDISTEIYQAGSTPC 
S2   ............................................................ 
S3   ............................................................ 
S4   ............................................................ 
S5   ............................................................ 
S6   ............................................................ 
S7   ............................................................ 
S8   ............................................................ 
S9   ............................................................ 
S10  ............................................................ 
S11  ............................................................ 
S12  ............................................................ 
S13  ............................................................ 
S14  ............................................................ 
S15  ............................................................ 
S16  ............------------------------------------------------ 
S17  ............................................................ 
S18  ............................................................ 
S19  ............................................................ 
S20  ............................................................ 
S21  ............................................................ 
S22  ............------------------------------------------------ 

             490       500       510       520       530       540     
     ....|....|....|....|....|....|....|....|....|....|....|....|
S1   NGVEGFNCYFPLQSYGFQPTNGVGYQPYRVVVLSFELLHAPATVCGPKKSTNLVKNKCVN 
S2   ............................................................ 
S3   ............................................................ 
S4   ............................................................ 
S5   ............................................................ 
S6   ............................................................ 
S7   ............................................................ 
S8   ............................................................ 
S9   ............................................................ 
S10  ............................................................ 
S11  ............................................................ 
S12  ............................................................ 
S13  ............................................................ 
S14  ............................................................ 
S15  ............................................................ 
S16  -------------------------------------------------........... 
S17  ............................................................ 
S18  ............................................................ 
S19  ............................................................ 
S20  ............................................................ 
S21  ............................................................ 
S22  -------------------------------------------------........... 

             550       560       570       580       590       600     
     ....|....|....|....|....|....|....|....|....|....|....|....|
S1   FNFNGLTGTGVLTESNKKFLPFQQFGRDIADTTDAVRDPQTLEILDITPCSFGGVSVITP 
S2   ............................................................ 
S3   ............................................................ 
S4   ............................................................ 
S5   ............................................................ 
S6   ............................................................ 
S7   ............................................................ 
S8   ............................................................ 
S9   ............................................................ 
S10  ............................................................ 
S11  ............................................................ 
S12  ............................................................ 
S13  ............................................................ 
S14  ............................................................ 
S15  ............................................................ 
S16  ............................................................ 
S17  ............................................................ 
S18  ............................................................ 
S19  ............................................................ 
S20  ............................................................ 
S21  ............................................................ 
S22  ............................................................ 

             610       620       630       640       650       660     
     ....|....|....|....|....|....|....|....|....|....|....|....|
S1   GTNTSNQVAVLYQDVNCTEVPVAIHADQLTPTWRVYSTGSNVFQTRAGCLIGAEHVNNSY 
S2   ............................................................ 
S3   ............................................................ 
S4   ............................................................ 
S5   ............................................................ 
S6   ............................................................ 
S7   ............................................................ 
S8   ............................................................ 
S9   ............................................................ 
S10  ............................................................ 
S11  .............G.............................................. 
S12  ............................................................ 
S13  ............................................................ 
S14  ............................................................ 
S15  ............................................................ 
S16  ............................................................ 
S17  .............G.............................................. 
S18  ............................................................ 
S19  ..............L............................................. 
S20  .............G.............................................. 
S21  ............................................................ 
S22  .............G.............................................. 

             670       680       690       700       710       720     
     ....|....|....|....|....|....|....|....|....|....|....|....|
S1   ECDIPIGAGICASYQTQTNSPRRARSVASQSIIAYTMSLGAENSVAYSNNSIAIPTNFTI 
S2   ............................................................ 
S3   ............................................................ 
S4   ............................................................ 
S5   ............................................................ 
S6   ............................................................ 
S7   ............................................................ 
S8   ............................................................ 
S9   ............................................................ 
S10  ............................................................ 
S11  ............................................................ 
S12  ............................................................ 
S13  ............................................................ 
S14  ............................................................ 
S15  ............................................................ 
S16  ............................................................ 
S17  ............................................................ 
S18  ............................................................ 
S19  ............................................................ 
S20  ............................................................ 
S21  ............................................................ 
S22  ............................................................ 

             730       740       750       760       770       780     
     ....|....|....|....|....|....|....|....|....|....|....|....|
S1   SVTTEILPVSMTKTSVDCTMYICGDSTECSNLLLQYGSFCTQLNRALTGIAVEQDKNTQE 
S2   ............................................................ 
S3   ............................................................ 
S4   ............................................................ 
S5   ............................................................ 
S6   ............................................................ 
S7   ............................................................ 
S8   ............................................................ 
S9   ............................................................ 
S10  ............................................................ 
S11  ............................................................ 
S12  ............................................................ 
S13  ............................................................ 
S14  ............................................................ 
S15  ............................................................ 
S16  ............................................................ 
S17  ............................................................ 
S18  ...............................F............................ 
S19  ............................................................ 
S20  ............................................................ 
S21  ............................................................ 
S22  ............................................................ 

             790       800       810       820       830       840     
     ....|....|....|....|....|....|....|....|....|....|....|....|
S1   VFAQVKQIYKTPPIKDFGGFNFSQILPDPSKPSKRSFIEDLLFNKVTLADAGFIKQYGDC 
S2   ................C........................................... 
S3   ............................................................ 
S4   ............................................................ 
S5   ............................................................ 
S6   ............................................................ 
S7   ............................................................ 
S8   ............................................................ 
S9   ............................................................ 
S10  ............................................................ 
S11  ............................................................ 
S12  ............................................................ 
S13  ............................................................ 
S14  ............................................................ 
S15  ............................................................ 
S16  ............................................................ 
S17  ............................................................ 
S18  ............................................................ 
S19  ............................................................ 
S20  ............................................................ 
S21  ............................................................ 
S22  ............................................................ 

             850       860       870       880       890       900     
     ....|....|....|....|....|....|....|....|....|....|....|....|
S1   LGDIAARDLICAQKFNGLTVLPPLLTDEMIAQYTSALLAGTITSGWTFGAGAALQIPFAM 
S2   ............................................................ 
S3   ............................................................ 
S4   ............................................................ 
S5   ............................................................ 
S6   ............................................................ 
S7   ............................................................ 
S8   ............................................................ 
S9   ............................................................ 
S10  ............................................................ 
S11  ............................................................ 
S12  ............................................................ 
S13  ...................QK....................................... 
S14  ............................................................ 
S15  ............................................................ 
S16  ............................................................ 
S17  ............................................................ 
S18  ............................................................ 
S19  ............................................................ 
S20  ............................................................ 
S21  ............................................................ 
S22  ............................................................ 

             910       920       930       940       950       960     
     ....|....|....|....|....|....|....|....|....|....|....|....|
S1   QMAYRFNGIGVTQNVLYENQKLIANQFNSAIGKIQDSLSSTASALGKLQDVVNQNAQALN 
S2   ............................................................ 
S3   ............................................................ 
S4   ............................................................ 
S5   ............................................................ 
S6   ............................................................ 
S7   ............................................................ 
S8   ............................................................ 
S9   ............................................................ 
S10  ............................................................ 
S11  ............................................................ 
S12  ............................................................ 
S13  ............................................................ 
S14  ............................................................ 
S15  ............................................................ 
S16  ............................................................ 
S17  ............................................................ 
S18  ............................................................ 
S19  ............................................................ 
S20  ............................................................ 
S21  ......................................F..................... 
S22  ............................................................ 

             970       980       990       1000      1010      1020    
     ....|....|....|....|....|....|....|....|....|....|....|....|
S1   TLVKQLSSNFGAISSVLNDILSRLDKVEAEVQIDRLITGRLQSLQTYVTQQLIRAAEIRA 
S2   ............................................................ 
S3   ............................................................ 
S4   ............................................................ 
S5   ............................................................ 
S6   ............................................................ 
S7   ............................................................ 
S8   ............................................................ 
S9   ............................................................ 
S10  ............................................................ 
S11  ............................................................ 
S12  ............................................................ 
S13  .........S.................................................. 
S14  ............................................................ 
S15  ............................................................ 
S16  ............................................................ 
S17  ............................................................ 
S18  ............................................................ 
S19  ............................................................ 
S20  ............................................................ 
S21  ............................................................ 
S22  ............................................................ 

             1030      1040      1050      1060      1070      1080    
     ....|....|....|....|....|....|....|....|....|....|....|....|
S1   SANLAATKMSECVLGQSKRVDFCGKGYHLMSFPQSAPHGVVFLHVTYVPAQEKNFTTAPA 
S2   ............................................................ 
S3   ............................................................ 
S4   ............................................................ 
S5   ............................................................ 
S6   ............................................................ 
S7   ............................................................ 
S8   ............................................................ 
S9   ............................................................ 
S10  ............................................................ 
S11  ............................................................ 
S12  ............................................................ 
S13  ............................................................ 
S14  ............................................................ 
S15  ............................................................ 
S16  ............................................................ 
S17  ............................................................ 
S18  ............................................................ 
S19  ............................................................ 
S20  ............................................................ 
S21  ............................................................ 
S22  ............................................................ 

             1090      1100      1110      1120      1130      1140    
     ....|....|....|....|....|....|....|....|....|....|....|....|
S1   ICHDGKAHFPREGVFVSNGTHWFVTQRNFYEPQIITTDNTFVSGNCDVVIGIVNNTVYDP 
S2   ............................................................ 
S3   ............................................................ 
S4   ............................................................ 
S5   ............................................................ 
S6   ............................................................ 
S7   ............................................................ 
S8   ................................................L........... 
S9   ............................................................ 
S10  ............................................................ 
S11  ............................................................ 
S12  ............................................................ 
S13  ............................................................ 
S14  ............................................................ 
S15  ............................................................ 
S16  ............................................................ 
S17  ............................................................ 
S18  ............................................................ 
S19  ............................................................ 
S20  ............................................................ 
S21  ............................................................ 
S22  ............................................................ 

             1150      1160      1170      1180      1190      1200    
     ....|....|....|....|....|....|....|....|....|....|....|....|
S1   LQPELDSFKEELDKYFKNHTSPDVDLGDISGINASVVNIQKEIDRLNEVAKNLNESLIDL 
S2   ............................................................ 
S3   ............................................................ 
S4   ............................................................ 
S5   ............................................................ 
S6   ............................................................ 
S7   ............................................................ 
S8   ............................................................ 
S9   ............................................................ 
S10  ............................................................ 
S11  ............................................................ 
S12  ..L......................................................... 
S13  ............................................................ 
S14  .............................Y.............................. 
S15  ............................................................ 
S16  ............................................................ 
S17  ............................................................ 
S18  ............................................................ 
S19  ............................................................ 
S20  ............................................................ 
S21  ............................................................ 
S22  ............................................................ 

             1210      1220      1230      1240      1250      1260    
     ....|....|....|....|....|....|....|....|....|....|....|....|
S1   QELGKYEQYIKWPWYIWLGFIAGLIAIVMVTIMLCCMTSCCSCLKGCCSCGSCCKFDEDD 
S2   ............................................................ 
S3   ............................................................ 
S4   ............................................................ 
S5   ............................................................ 
S6   ............................................................ 
S7   ............................................................ 
S8   ............................................................ 
S9   ............................................................ 
S10  ............................................................ 
S11  ............................................................ 
S12  ............................................................ 
S13  ............................................................ 
S14  ............................................................ 
S15  ............................................................ 
S16  ............................................................ 
S17  ............................................................ 
S18  ............................................................ 
S19  ............................................................ 
S20  ............................................................ 
S21  ............................................................ 
S22  ............................................................ 

             1270 
     ....|....|...
S1   SEPVLKGVKLHYT 
S2   ............. 
S3   ............. 
S4   ............. 
S5   ............. 
S6   ............. 
S7   ............. 
S8   .G........... 
S9   ............. 
S10  ............. 
S11  ............. 
S12  ............. 
S13  ............. 
S14  ..L.......... 
S15  ............. 
S16  ............. 
S17  ............. 
S18  ............. 
S19  ............. 
S20  ............. 
S21  ............. 
S22  ............. 
