# Supplementary material for: First computational design using lambda-superstrings and in vivo validation of SARS-CoV-2 vaccine
Source: Sci Rep. 2022 Apr 19;12:6410. doi: 10.1038/s41598-022-09615-w (PMC9016385; doi:10.1038/s41598-022-09615-w)
Supplement: Supplementary file 3 — Supplementary Information 3. [file 41598_2022_9615_MOESM3_ESM.docx]

**Optimal weighted λ–superstring, λ values and VaxiJen overall prediction for lengths between 9 and 280 amino acids.**

Column 1, the number of amino acids in the CVs; column 2, the value of **λ**; column 3, the VaxiJen overall prediction for antigenicity; column 4, the peptides whose union forms the **λ** -superstring. The candidates whose global overall prediction (the one of the string obtained joining their constituent peptides) is over 0.4 are colored in green.

| Length | Lambda | Prediction | Sequence |
| --- | --- | --- | --- |
| 9 | 0.75 | -0.2311 | 1:IRGWIFGTT(NTD) |
| 10 | 1.49 | -0.8187 | 1:SNIIRGWIFG(NTD) |
| 11 | 2.24 | -0.3450 | 1:NIIRGWIFGTT(NTD) |
| 12 | 2.99 | -0.3537 | 1:SNIIRGWIFGTT(NTD) |
| 13 | 3.7 | -0.5324 | 1:KSNIIRGWIFGTT(NTD) |
| 14 | 4.37 | -0.5608 | 1:KSNIIRGWIFGTTL(NTD) |
| 15 | 4.96 | -0.3688 | 1:EKSNIIRGWIFGTTL(NTD) |
| 16 | 5.53 | -0.2956 | 1:EKSNIIRGWIFGTTLD(NTD) |
| 17 | 6.05 | -0.3730 | 1:EKSNIIRGWIFGTTLDS(NTD) |
| 18 | 6.48 | -0.2974 | 1:TEKSNIIRGWIFGTTLDS(NTD) |
| 19 | 6.9 | 0.0025 | 1:TEKSNIIRGWIFGTTLDSK(NTD) |
| 20 | 7.23 | 0.0044 | 1:STEKSNIIRGWIFGTTLDSK(NTD) |
| 21 | 7.54 | 0.0359 | 1:ASTEKSNIIRGWIFGTTLDSK(NTD) |
| 22 | 8.03 | 0.5545 | 1:STQDLFLPFFSNVTWFHAIHVS(NTD) |
| 23 | 8.54 | 0.3591 | 1:STQDLFLPFFSNVTWFHAIHVSG(NTD) |
| 24 | 9.02 | 0.4796 | 1:STQDLFLPFFSNVTWFHAIHVSGT(NTD) |
| 25 | 9.47 | 0.2571 | 1:DGKAHFPREGVFVSNGTHWFVTQRN(CD) |
| 26 | 10.02 | 0.3869 | 1:FVSNGTHWFVTQRNFYEPQIITTDNT(CD) |
| 27 | 10.54 | 0.3742 | 1:FVSNGTHWFVTQRNFYEPQIITTDNTF(CD) |
| 28 | 11.06 | 0.3592 | 1:FVSNGTHWFVTQRNFYEPQIITTDNTFV(CD) |
| 29 | 11.6 | 0.3892 | 1:FVSNGTHWFVTQRNFYEPQIITTDNTFVS  (CD) |
| 30 | 12.07 | 0.3131 | 1:FVSNGTHWFVTQRNFYEPQIITTDNTFVSG  (CD) |
| 31 | 12.56 | 0.2632 | 1:QPTESIVRFPNITNLCPFGEVFNATRFASVY  (RBD) |
| 32 | 13.05 | 0.3711 | 1:VQPTESIVRFPNITNLCPFGEVFNATRFASVY(RBD) |
| 33 | 13.52 | 0.3698 | 1:VQPTESIVRFPNITNLCPFGEVFNATRFASVYA(RBD) |
| 34 | 14 | 0.3097 | 1:KAHFPREGVFVSNGTHWFVTQRNFYEPQIITTDN(CD) |
| 35 | 14.59 | 0.3260 | 1:KAHFPREGVFVSNGTHWFVTQRNFYEPQIITTDNT  (CD) |
| 36 | 15.17 | 0.3278 | 1:GKAHFPREGVFVSNGTHWFVTQRNFYEPQIITTDNT  (CD) |
| 37 | 15.68 | 0.3198 | 1:GKAHFPREGVFVSNGTHWFVTQRNFYEPQIITTDNTF  (CD) |
| 38 | 16.21 | 0.3107 | 1:GKAHFPREGVFVSNGTHWFVTQRNFYEPQIITTDNTFV  (CD) |
| 39 | 16.74 | 0.3341 | 1:GKAHFPREGVFVSNGTHWFVTQRNFYEPQIITTDNTFVS  (CD) |
| 40 | 17.24 | 0.3078 | 1:DGKAHFPREGVFVSNGTHWFVTQRNFYEPQIITTDNTFVS(CD) |
| 41 | 17.72 | 0.2556 | 1:DGKAHFPREGVFVSNGTHWFVTQRNFYEPQIITTDNTFVSG(CD) |
| 42 | 18.18 | 0.2660 | 1:DGKAHFPREGVFVSNGTHWFVTQRNFYEPQIITTDNTFVSGN(CD) |
| 43 | 18.62 | 0.2511 | 1:DGKAHFPREGVFVSNGTHWFVTQRNFYEPQIITTDNTFVSGNC(CD) |
| 44 | 19.01 | 0.2414 | 1:HDGKAHFPREGVFVSNGTHWFVTQRNFYEPQIITTDNTFVSGNC(CD) |
| 45 | 19.4 | 0.2132 | 1:CHDGKAHFPREGVFVSNGTHWFVTQRNFYEPQIITTDNTFVSGNC(CD) |
| 46 | 19.76 | 0.1944 | 1:ICHDGKAHFPREGVFVSNGTHWFVTQRNFYEPQIITTDNTFVSGNC(CD) |
| 47 | 20.16 | 0.2598 | 1:TAPAICHDGKAHFPREGVFVSNGTHWFVTQRNFYEPQIITTDNTFVS(CD) |
| 48 | 20.69 | 0.2454 | 1:KNFTTAPAICHDGKAHFPREGVFVSNGTHWFVTQRNFYEPQIITTDNT(CD) |
| 49 | 21.24 | 0.2717 | 1:EKNFTTAPAICHDGKAHFPREGVFVSNGTHWFVTQRNFYEPQIITTDNT(CD) |
| 50 | 21.76 | 0.2667 | 1:EKNFTTAPAICHDGKAHFPREGVFVSNGTHWFVTQRNFYEPQIITTDNTF(CD) |
| 51 | 22.28 | 0.2612 | 1:EKNFTTAPAICHDGKAHFPREGVFVSNGTHWFVTQRNFYEPQIITTDNTFV(CD) |
| 52 | 22.82 | 0.2796 | 1:EKNFTTAPAICHDGKAHFPREGVFVSNGTHWFVTQRNFYEPQIITTDNTFVS(CD) |
| 53 | 23.32 | 0.3250 | 1:QEKNFTTAPAICHDGKAHFPREGVFVSNGTHWFVTQRNFYEPQIITTDNTFVS(CD) |
| 54 | 23.79 | 0.2857 | 1:QEKNFTTAPAICHDGKAHFPREGVFVSNGTHWFVTQRNFYEPQIITTDNTFVSG(CD) |
| 55 | 24.28 | 0.2378 | 1:FFSNVTWFHAIHVSGTNGTKRFDNPVLPFNDGVYFASTEKSNIIRGWIFGTTLDS(NTD) |
| 56 | 24.77 | 0.3126 | 1:LPFFSNVTWFHAIHVSGTNGTKRFDNPVLPFNDGVYFASTEKSNIIRGWIFGTTLD(NTD) |
| 57 | 25.28 | 0.2794 | 1:LPFFSNVTWFHAIHVSGTNGTKRFDNPVLPFNDGVYFASTEKSNIIRGWIFGTTLDS(NTD) |
| 58 | 25.75 | 0.2722 | 1:FLPFFSNVTWFHAIHVSGTNGTKRFDNPVLPFNDGVYFASTEKSNIIRGWIFGTTLDS(NTD) |
| 59 | 26.21 | 0.2321 | 1:LFLPFFSNVTWFHAIHVSGTNGTKRFDNPVLPFNDGVYFASTEKSNIIRGWIFGTTLDS(NTD) |
| 60 | 26.78 | 0.2699 | 1:QDLFLPFFSNVTWFHAIHVSGTNGTKRFDNPVLPFNDGVYFASTEKSNIIRGWIFGTTLD(NTD) |
| 61 | 27.31 | 0.2767 | 1:TQDLFLPFFSNVTWFHAIHVSGTNGTKRFDNPVLPFNDGVYFASTEKSNIIRGWIFGTTLD(NTD) |
| 62 | 27.84 | 0.2893 | 1:STQDLFLPFFSNVTWFHAIHVSGTNGTKRFDNPVLPFNDGVYFASTEKSNIIRGWIFGTTLD  (NTD) |
| 63 | 28.35 | 0.2598 | 1:STQDLFLPFFSNVTWFHAIHVSGTNGTKRFDNPVLPFNDGVYFASTEKSNIIRGWIFGTTLDS  (NTD) |
| 64 | 28.77 | 0.3308 | 1:STQDLFLPFFSNVTWFHAIHVSGTNGTKRFDNPVLPFNDGVYFASTEKSNIIRGWIFGTTLDSK  (NTD) |
| 65 | 29.06 | 0.3140 | 1:STQDLFLPFFSNVTWFHAIHVSGTNGTKRFDNPVLPFNDGVYFASTEKSNIIRGWIFGTTLDSKT  (NTD) |
| 66 | 29.29 | 0.3344 | 1:STQDLFLPFFSNVTWFHAIHVSGTNGTKRFDNPVLPFNDGVYFASTEKSNIIRGWIFGTTLDSKTQ(NTD) |
| 67 | 29.79 | 0.4214 | 1:QSAPHGVVFLHVTYVPAQEKNFTTAPAICHDGKAHFPREGVFVSNGTHWFVTQRNFYEPQIITTDNT(CD) |
| 68 | 30.3 | 0.4153 | 1:QSAPHGVVFLHVTYVPAQEKNFTTAPAICHDGKAHFPREGVFVSNGTHWFVTQRNFYEPQIITTDNTF(CD) |
| 69 | 30.83 | 0.4090 | 1:QSAPHGVVFLHVTYVPAQEKNFTTAPAICHDGKAHFPREGVFVSNGTHWFVTQRNFYEPQIITTDNTFV(CD) |
| 70 | 31.36 | 0.4203 | 1:QSAPHGVVFLHVTYVPAQEKNFTTAPAICHDGKAHFPREGVFVSNGTHWFVTQRNFYEPQIITTDNTFVS(CD) |
| 71 | 31.84 | 0.3893 | 1:QSAPHGVVFLHVTYVPAQEKNFTTAPAICHDGKAHFPREGVFVSNGTHWFVTQRNFYEPQIITTDNTFVSG(CD) |
| 72 | 32.3 | 0.3930 | 1:QSAPHGVVFLHVTYVPAQEKNFTTAPAICHDGKAHFPREGVFVSNGTHWFVTQRNFYEPQIITTDNTFVSGN(CD) |
| 73 | 32.75 | 0.3826 | 1:PQSAPHGVVFLHVTYVPAQEKNFTTAPAICHDGKAHFPREGVFVSNGTHWFVTQRNFYEPQIITTDNTFVSGN(CD) |
| 74 | 33.19 | 0.3730 | 1:PQSAPHGVVFLHVTYVPAQEKNFTTAPAICHDGKAHFPREGVFVSNGTHWFVTQRNFYEPQIITTDNTFVSGNC(CD) |
| 75 | 33.56 | 0.3458 | 1:FPQSAPHGVVFLHVTYVPAQEKNFTTAPAICHDGKAHFPREGVFVSNGTHWFVTQRNFYEPQIITTDNTFVSGNC(CD) |
| 76 | 33.92 | 0.3364 | 1:FPQSAPHGVVFLHVTYVPAQEKNFTTAPAICHDGKAHFPREGVFVSNGTHWFVTQRNFYEPQIITTDNTFVSGNCD(CD) |
| 77 | 34.24 | 0.3248 | 1:FPQSAPHGVVFLHVTYVPAQEKNFTTAPAICHDGKAHFPREGVFVSNGTHWFVTQRNFYEPQIITTDNTFVSGNCDV(CD) |
| 78 | 34.51 | 0.3250 | 1:SFPQSAPHGVVFLHVTYVPAQEKNFTTAPAICHDGKAHFPREGVFVSNGTHWFVTQRNFYEPQIITTDNTFVSGNCDV(CD) |
| 79 | 34.79 | 0.3380 | 1:MSFPQSAPHGVVFLHVTYVPAQEKNFTTAPAICHDGKAHFPREGVFVSNGTHWFVTQRNFYEPQIITTDNTFVSGNCDV(CD) |
| 80 | 35.14 | 0.3279 | 1:HLMSFPQSAPHGVVFLHVTYVPAQEKNFTTAPAICHDGKAHFPREGVFVSNGTHWFVTQRNFYEPQIITTDNTFVSGNCD(CD) |
| 81 | 35.46 | 0.3171 | 1:HLMSFPQSAPHGVVFLHVTYVPAQEKNFTTAPAICHDGKAHFPREGVFVSNGTHWFVTQRNFYEPQIITTDNTFVSGNCDV(CD) |
| 82 | 35.8 | 0.2706 | 1:DGKAHFPREGVFVSNGTHWFVTQRNFYEPQIITTDNTFVSG(CD)  2:TNGTKRFDNPVLPFNDGVYFASTEKSNIIRGWIFGTTLDSK(NTD) |
| 83 | 36.19 | 0.2654 | 1:HDGKAHFPREGVFVSNGTHWFVTQRNFYEPQIITTDNTFVSG(CD)  2:TNGTKRFDNPVLPFNDGVYFASTEKSNIIRGWIFGTTLDSK(NTD) |
| 84 | 36.58 | 0.2505 | 1:CHDGKAHFPREGVFVSNGTHWFVTQRNFYEPQIITTDNTFVSG(CD)  2:TNGTKRFDNPVLPFNDGVYFASTEKSNIIRGWIFGTTLDSK(NTD) |
| 85 | 36.94 | 0.2402 | 1:ICHDGKAHFPREGVFVSNGTHWFVTQRNFYEPQIITTDNTFVSG(CD)  2:TNGTKRFDNPVLPFNDGVYFASTEKSNIIRGWIFGTTLDSK(NTD) |
| 86 | 37.34 | 0.2613 | 1:EKSNIIRGWIFGTTLDS(NTD)  2:APHGVVFLHVTYVPAQEKNFTTAPAICHDGKAHFPREGVFVSNGTHWFVTQRNFYEPQIITTDNTFVSG(CD) |
| 87 | 37.84 | 0.3043 | 1:FVSNGTHWFVTQRNFYEPQIITTDNT(CD)  2:QDLFLPFFSNVTWFHAIHVSGTNGTKRFDNPVLPFNDGVYFASTEKSNIIRGWIFGTTLDS(NTD) |
| 88 | 38.3 | 0.1983 | 1:TAPAICHDGKAHFPREGVFVSNGTHWFVTQRNFYEPQIITTDNTFVSG(CD)  2:TNGTKRFDNPVLPFNDGVYFASTEKSNIIRGWIFGTTLDS(NTD) |
| 89 | 38.87 | 0.2950 | 1:KAHFPREGVFVSNGTHWFVTQRNFYEPQIITTDNTF(CD)  2:FSNVTWFHAIHVSGTNGTKRFDNPVLPFNDGVYFASTEKSNIIRGWIFGTTLD(NTD) |
| 90 | 39.45 | 0.2964 | 1:GKAHFPREGVFVSNGTHWFVTQRNFYEPQIITTDNTF(CD)  2:FSNVTWFHAIHVSGTNGTKRFDNPVLPFNDGVYFASTEKSNIIRGWIFGTTLD(NTD) |
| 91 | 39.96 | 0.2760 | 1:GKAHFPREGVFVSNGTHWFVTQRNFYEPQIITTDNTF(CD)  2:FSNVTWFHAIHVSGTNGTKRFDNPVLPFNDGVYFASTEKSNIIRGWIFGTTLDS(NTD) |
| 92 | 40.47 | 0.2729 | 1:FFSNVTWFHAIHVSGTNGTKRFDNPVLPFNDGVYFASTEKSNIIRGWIFGTTLD(NTD)  2:GKAHFPREGVFVSNGTHWFVTQRNFYEPQIITTDNTFV(CD) |
| 93 | 41.01 | 0.2830 | 1:FFSNVTWFHAIHVSGTNGTKRFDNPVLPFNDGVYFASTEKSNIIRGWIFGTTLD(NTD)  2:GKAHFPREGVFVSNGTHWFVTQRNFYEPQIITTDNTFVS(CD) |
| 94 | 41.49 | 0.3018 | 1:PFFSNVTWFHAIHVSGTNGTKRFDNPVLPFNDGVYFASTEKSNIIRGWIFGTTLD(NTD)  2:GKAHFPREGVFVSNGTHWFVTQRNFYEPQIITTDNTFVS(CD) |
| 95 | 42.01 | 0.3067 | 1:LPFFSNVTWFHAIHVSGTNGTKRFDNPVLPFNDGVYFASTEKSNIIRGWIFGTTLD(NTD)  2:GKAHFPREGVFVSNGTHWFVTQRNFYEPQIITTDNTFVS(CD) |
| 96 | 42.48 | 0.3023 | 1:FLPFFSNVTWFHAIHVSGTNGTKRFDNPVLPFNDGVYFASTEKSNIIRGWIFGTTLD(NTD)  2:GKAHFPREGVFVSNGTHWFVTQRNFYEPQIITTDNTFVS(CD) |
| 97 | 42.99 | 0.2931 | 1:GKAHFPREGVFVSNGTHWFVTQRNFYEPQIITTDNT(CD)  2:QDLFLPFFSNVTWFHAIHVSGTNGTKRFDNPVLPFNDGVYFASTEKSNIIRGWIFGTTLDS(NTD) |
| 98 | 43.51 | 0.2898 | 1:STQDLFLPFFSNVTWFHAIHVSGTNGTKRFDNPVLPFNDGVYFASTEKSNIIRGWIFGTTLD  (NTD)  2:GKAHFPREGVFVSNGTHWFVTQRNFYEPQIITTDNT(CD) |
| 99 | 44.02 | 0.2869 | 1:STQDLFLPFFSNVTWFHAIHVSGTNGTKRFDNPVLPFNDGVYFASTEKSNIIRGWIFGTTLD  (NTD)  2:GKAHFPREGVFVSNGTHWFVTQRNFYEPQIITTDNTF(CD) |
| 100 | 44.58 | 0.3080 | 1:GKAHFPREGVFVSNGTHWFVTQRNFYEPQIITTDNTFVS(CD)  2:TQDLFLPFFSNVTWFHAIHVSGTNGTKRFDNPVLPFNDGVYFASTEKSNIIRGWIFGTTLD(NTD) |
| 101 | 45.1 | 0.2895 | 1:GKAHFPREGVFVSNGTHWFVTQRNFYEPQIITTDNTFVS(CD)  2:TQDLFLPFFSNVTWFHAIHVSGTNGTKRFDNPVLPFNDGVYFASTEKSNIIRGWIFGTTLDS  (NTD) |
| 102 | 45.6 | 0.2798 | 1:DGKAHFPREGVFVSNGTHWFVTQRNFYEPQIITTDNTFVS(CD)  2:TQDLFLPFFSNVTWFHAIHVSGTNGTKRFDNPVLPFNDGVYFASTEKSNIIRGWIFGTTLDS  (NTD) |
| 103 | 46.02 | 0.2766 | 1:STQDLFLPFFSNVTWFHAIHVSGTNGTKRFDNPVLPFNDGVYFASTEKSNIIRGWIFGTTLD  (NTD)  2:GKAHFPREGVFVSNGTHWFVTQRNFYEPQIITTDNTFVSGN(CD) |
| 104 | 46.46 | 0.2710 | 1:STQDLFLPFFSNVTWFHAIHVSGTNGTKRFDNPVLPFNDGVYFASTEKSNIIRGWIFGTTLD  (NTD)  2:GKAHFPREGVFVSNGTHWFVTQRNFYEPQIITTDNTFVSGNC(CD) |
| 105 | 46.82 | 0.2649 | 1:STQDLFLPFFSNVTWFHAIHVSGTNGTKRFDNPVLPFNDGVYFASTEKSNIIRGWIFGTTLD  (NTD)  2:GKAHFPREGVFVSNGTHWFVTQRNFYEPQIITTDNTFVSGNCD(CD) |
| 106 | 47.25 | 0.2723 | 1:STQDLFLPFFSNVTWFHAIHVSGTNGTKRFDNPVLPFNDGVYFASTEKSNIIRGWIFGTT(NTD)  2:APAICHDGKAHFPREGVFVSNGTHWFVTQRNFYEPQIITTDNTFVS(CD) |
| 107 | 47.73 | 0.2534 | 1:STQDLFLPFFSNVTWFHAIHVSGTNGTKRFDNPVLPFNDGVYFASTEKSNIIRGWIFGTT(NTD)  2:APAICHDGKAHFPREGVFVSNGTHWFVTQRNFYEPQIITTDNTFVSG(CD) |
| 108 | 48.19 | 0.2570 | 1:STQDLFLPFFSNVTWFHAIHVSGTNGTKRFDNPVLPFNDGVYFASTEKSNIIRGWIFGTT(NTD)  2:APAICHDGKAHFPREGVFVSNGTHWFVTQRNFYEPQIITTDNTFVSGN(CD) |
| 109 | 48.63 | 0.2518 | 1:STQDLFLPFFSNVTWFHAIHVSGTNGTKRFDNPVLPFNDGVYFASTEKSNIIRGWIFGTT(NTD)  2:APAICHDGKAHFPREGVFVSNGTHWFVTQRNFYEPQIITTDNTFVSGNC(CD) |
| 110 | 49.06 | 0.2737 | 1:EKNFTTAPAICHDGKAHFPREGVFVSNGTHWFVTQRNFYEPQIITTDNT(CD)  2:QDLFLPFFSNVTWFHAIHVSGTNGTKRFDNPVLPFNDGVYFASTEKSNIIRGWIFGTTLDS(NTD) |
| 111 | 49.56 | 0.2949 | 1:NFTTAPAICHDGKAHFPREGVFVSNGTHWFVTQRNFYEPQIITTDNTFVS(CD)  2:TQDLFLPFFSNVTWFHAIHVSGTNGTKRFDNPVLPFNDGVYFASTEKSNIIRGWIFGTTLD(NTD) |
| 112 | 50.11 | 0.2766 | 1:KNFTTAPAICHDGKAHFPREGVFVSNGTHWFVTQRNFYEPQIITTDNTFVS(CD)  2:TQDLFLPFFSNVTWFHAIHVSGTNGTKRFDNPVLPFNDGVYFASTEKSNIIRGWIFGTTLD(NTD) |
| 113 | 50.66 | 0.2874 | 1:EKNFTTAPAICHDGKAHFPREGVFVSNGTHWFVTQRNFYEPQIITTDNTFVS(CD)  2:TQDLFLPFFSNVTWFHAIHVSGTNGTKRFDNPVLPFNDGVYFASTEKSNIIRGWIFGTTLD(NTD) |
| 114 | 51.17 | 0.2713 | 1:EKNFTTAPAICHDGKAHFPREGVFVSNGTHWFVTQRNFYEPQIITTDNTFVS(CD)  2:TQDLFLPFFSNVTWFHAIHVSGTNGTKRFDNPVLPFNDGVYFASTEKSNIIRGWIFGTTLDS  (NTD) |
| 115 | 51.67 | 0.2918 | 1:QEKNFTTAPAICHDGKAHFPREGVFVSNGTHWFVTQRNFYEPQIITTDNTFVS(CD)  2:TQDLFLPFFSNVTWFHAIHVSGTNGTKRFDNPVLPFNDGVYFASTEKSNIIRGWIFGTTLDS  (NTD) |
| 116 | 52.09 | 0.3300 | 1:QEKNFTTAPAICHDGKAHFPREGVFVSNGTHWFVTQRNFYEPQIITTDNTFVS(CD)  2:TQDLFLPFFSNVTWFHAIHVSGTNGTKRFDNPVLPFNDGVYFASTEKSNIIRGWIFGTTLDSK  (NTD) |
| 117 | 52.49 | 0.3297 | 1:AQEKNFTTAPAICHDGKAHFPREGVFVSNGTHWFVTQRNFYEPQIITTDNTFVS(CD)  2:TQDLFLPFFSNVTWFHAIHVSGTNGTKRFDNPVLPFNDGVYFASTEKSNIIRGWIFGTTLDSK  (NTD) |
| 118 | 52.88 | 0.3222 | 1:PAQEKNFTTAPAICHDGKAHFPREGVFVSNGTHWFVTQRNFYEPQIITTDNTFVS(CD)  2:TQDLFLPFFSNVTWFHAIHVSGTNGTKRFDNPVLPFNDGVYFASTEKSNIIRGWIFGTTLDSK  (NTD) |
| 119 | 53.21 | 0.3307 | 1:VPAQEKNFTTAPAICHDGKAHFPREGVFVSNGTHWFVTQRNFYEPQIITTDNTFVS(CD)  2:TQDLFLPFFSNVTWFHAIHVSGTNGTKRFDNPVLPFNDGVYFASTEKSNIIRGWIFGTTLDSK  (NTD) |
| 120 | 53.54 | 0.3181 | 1:STQDLFLPFFSNVTWFHAIHVSGTNGTKRFDNPVLPFNDGVYFASTEKSNIIRGWIFGTTLDSKTQ(NTD)  2:EKNFTTAPAICHDGKAHFPREGVFVSNGTHWFVTQRNFYEPQIITTDNTFVSGN(CD) |
| 121 | 54.07 | 0.3557 | 1:QSAPHGVVFLHVTYVPAQEKNFTTAPAICHDGKAHFPREGVFVSNGTHWFVTQRNFYEPQIITTDNTF(CD)  2:FSNVTWFHAIHVSGTNGTKRFDNPVLPFNDGVYFASTEKSNIIRGWIFGTTLD(NTD) |
| 122 | 54.58 | 0.3401 | 1:QSAPHGVVFLHVTYVPAQEKNFTTAPAICHDGKAHFPREGVFVSNGTHWFVTQRNFYEPQIITTDNTF(CD)  2:FSNVTWFHAIHVSGTNGTKRFDNPVLPFNDGVYFASTEKSNIIRGWIFGTTLDS(NTD) |
| 123 | 55.1 | 0.3414 | 1:FFSNVTWFHAIHVSGTNGTKRFDNPVLPFNDGVYFASTEKSNIIRGWIFGTTLDS(NTD)  2:APHGVVFLHVTYVPAQEKNFTTAPAICHDGKAHFPREGVFVSNGTHWFVTQRNFYEPQIITTDNTFVS(CD) |
| 124 | 55.58 | 0.3551 | 1:PFFSNVTWFHAIHVSGTNGTKRFDNPVLPFNDGVYFASTEKSNIIRGWIFGTTLDS(NTD)  2:APHGVVFLHVTYVPAQEKNFTTAPAICHDGKAHFPREGVFVSNGTHWFVTQRNFYEPQIITTDNTFVS(CD) |
| 125 | 56.1 | 0.3585 | 1:LPFFSNVTWFHAIHVSGTNGTKRFDNPVLPFNDGVYFASTEKSNIIRGWIFGTTLDS(NTD)  2:APHGVVFLHVTYVPAQEKNFTTAPAICHDGKAHFPREGVFVSNGTHWFVTQRNFYEPQIITTDNTFVS(CD) |
| 126 | 56.57 | 0.3547 | 1:FLPFFSNVTWFHAIHVSGTNGTKRFDNPVLPFNDGVYFASTEKSNIIRGWIFGTTLDS(NTD)  2:APHGVVFLHVTYVPAQEKNFTTAPAICHDGKAHFPREGVFVSNGTHWFVTQRNFYEPQIITTDNTFVS(CD) |
| 127 | 57.09 | 0.3649 | 1:QSAPHGVVFLHVTYVPAQEKNFTTAPAICHDGKAHFPREGVFVSNGTHWFVTQRNFYEPQIITTDNT(CD)  2:QDLFLPFFSNVTWFHAIHVSGTNGTKRFDNPVLPFNDGVYFASTEKSNIIRGWIFGTTLD(NTD) |
| 128 | 57.61 | 0.3500 | 1:QSAPHGVVFLHVTYVPAQEKNFTTAPAICHDGKAHFPREGVFVSNGTHWFVTQRNFYEPQIITTDNT(CD)  2:QDLFLPFFSNVTWFHAIHVSGTNGTKRFDNPVLPFNDGVYFASTEKSNIIRGWIFGTTLDS(NTD) |
| 129 | 58.12 | 0.3561 | 1:APHGVVFLHVTYVPAQEKNFTTAPAICHDGKAHFPREGVFVSNGTHWFVTQRNFYEPQIITTDNTFVS(CD)  2:TQDLFLPFFSNVTWFHAIHVSGTNGTKRFDNPVLPFNDGVYFASTEKSNIIRGWIFGTTLD(NTD) |
| 130 | 58.66 | 0.3508 | 1:SAPHGVVFLHVTYVPAQEKNFTTAPAICHDGKAHFPREGVFVSNGTHWFVTQRNFYEPQIITTDNTFVS(CD)  2:TQDLFLPFFSNVTWFHAIHVSGTNGTKRFDNPVLPFNDGVYFASTEKSNIIRGWIFGTTLD(NTD) |
| 131 | 59.2 | 0.3600 | 1:QSAPHGVVFLHVTYVPAQEKNFTTAPAICHDGKAHFPREGVFVSNGTHWFVTQRNFYEPQIITTDNTFVS(CD)  2:TQDLFLPFFSNVTWFHAIHVSGTNGTKRFDNPVLPFNDGVYFASTEKSNIIRGWIFGTTLD(NTD) |
| 132 | 59.72 | 0.3455 | 1:QSAPHGVVFLHVTYVPAQEKNFTTAPAICHDGKAHFPREGVFVSNGTHWFVTQRNFYEPQIITTDNTFVS(CD)  2:TQDLFLPFFSNVTWFHAIHVSGTNGTKRFDNPVLPFNDGVYFASTEKSNIIRGWIFGTTLDS  (NTD) |
| 133 | 60.17 | 0.3403 | 1:PQSAPHGVVFLHVTYVPAQEKNFTTAPAICHDGKAHFPREGVFVSNGTHWFVTQRNFYEPQIITTDNTFVS(CD)  2:TQDLFLPFFSNVTWFHAIHVSGTNGTKRFDNPVLPFNDGVYFASTEKSNIIRGWIFGTTLDS  (NTD) |
| 134 | 60.59 | 0.3729 | 1:PQSAPHGVVFLHVTYVPAQEKNFTTAPAICHDGKAHFPREGVFVSNGTHWFVTQRNFYEPQIITTDNTFVS(CD)  2:TQDLFLPFFSNVTWFHAIHVSGTNGTKRFDNPVLPFNDGVYFASTEKSNIIRGWIFGTTLDSK  (NTD) |
| 135 | 60.96 | 0.3580 | 1:FPQSAPHGVVFLHVTYVPAQEKNFTTAPAICHDGKAHFPREGVFVSNGTHWFVTQRNFYEPQIITTDNTFVS(CD)  2:TQDLFLPFFSNVTWFHAIHVSGTNGTKRFDNPVLPFNDGVYFASTEKSNIIRGWIFGTTLDSK  (NTD) |
| 136 | 61.3 | 0.3724 | 1:STQDLFLPFFSNVTWFHAIHVSGTNGTKRFDNPVLPFNDGVYFASTEKSNIIRGWIFGTTLDSKTQS(NTD)  2:APHGVVFLHVTYVPAQEKNFTTAPAICHDGKAHFPREGVFVSNGTHWFVTQRNFYEPQIITTDNTFVSG(CD) |
| 137 | 61.76 | 0.3744 | 1:STQDLFLPFFSNVTWFHAIHVSGTNGTKRFDNPVLPFNDGVYFASTEKSNIIRGWIFGTTLDSKTQS(NTD)  2:APHGVVFLHVTYVPAQEKNFTTAPAICHDGKAHFPREGVFVSNGTHWFVTQRNFYEPQIITTDNTFVSGN(CD) |
| 138 | 62.2 | 0.3695 | 1:STQDLFLPFFSNVTWFHAIHVSGTNGTKRFDNPVLPFNDGVYFASTEKSNIIRGWIFGTTLDSKTQS(NTD)  2:APHGVVFLHVTYVPAQEKNFTTAPAICHDGKAHFPREGVFVSNGTHWFVTQRNFYEPQIITTDNTFVSGNC(CD) |
| 139 | 62.56 | 0.3642 | 1:STQDLFLPFFSNVTWFHAIHVSGTNGTKRFDNPVLPFNDGVYFASTEKSNIIRGWIFGTTLDSKTQS(NTD)  2:APHGVVFLHVTYVPAQEKNFTTAPAICHDGKAHFPREGVFVSNGTHWFVTQRNFYEPQIITTDNTFVSGNCD(CD) |
| 140 | 62.88 | 0.3578 | 1:STQDLFLPFFSNVTWFHAIHVSGTNGTKRFDNPVLPFNDGVYFASTEKSNIIRGWIFGTTLDSKTQS(NTD)  2:APHGVVFLHVTYVPAQEKNFTTAPAICHDGKAHFPREGVFVSNGTHWFVTQRNFYEPQIITTDNTFVSGNCDV(CD) |
| 141 | 63.01 | 0.3140 | 1:STQDLFLPFFSNVTWFHAIHVSGTNGTKRFDNPVLPFNDGVYFASTEKSNIIRGWIFGTTLDSK  (NTD)  2:FPQSAPHGVVFLHVTYVPAQEKNFTTAPAICHDGKAHFPREGVFVSNGTHWFVTQRNFYEPQIITTDNTFVSGNCDV(CD) |
| 142 | 63.3 | 0.3307 | 1:FPQSAPHGVVFLHVTYVPAQEKNFTTAPAICHDGKAHFPREGVFVSNGTHWFVTQRNFYEPQIITTDNTFVSGNCDV(CD)  2:STQDLFLPFFSNVTWFHAIHVSGTNGTKRFDNPVLPFNDGVYFASTEKSNIIRGWIFGTTLDSKT  (NTD) |
| 143 | 63.65 | 0.3453 | 1:STQDLFLPFFSNVTWFHAIHVSGTNGTKRFDNPVLPFNDGVYFASTEKSNIIRGWIFGTTLDSKTQS(NTD)  2:FPQSAPHGVVFLHVTYVPAQEKNFTTAPAICHDGKAHFPREGVFVSNGTHWFVTQRNFYEPQIITTDNTFVSGNCD(CD) |
| 144 | 63.97 | 0.3392 | 1:STQDLFLPFFSNVTWFHAIHVSGTNGTKRFDNPVLPFNDGVYFASTEKSNIIRGWIFGTTLDSKTQS(NTD)  2:FPQSAPHGVVFLHVTYVPAQEKNFTTAPAICHDGKAHFPREGVFVSNGTHWFVTQRNFYEPQIITTDNTFVSGNCDV(CD) |
| 145 | 64.32 | 0.3381 | 1:STQDLFLPFFSNVTWFHAIHVSGTNGTKRFDNPVLPFNDGVYFASTEKSNIIRGWIFGTTLDSK  (NTD)  2:GYHLMSFPQSAPHGVVFLHVTYVPAQEKNFTTAPAICHDGKAHFPREGVFVSNGTHWFVTQRNFYEPQIITTDNTFVSGNC(CD) |
| 146 | 64.69 | 0.3111 | 1:STQDLFLPFFSNVTWFHAIHVSGTNGTKRFDNPVLPFNDGVYFASTEKSNIIRGWIFGTTLD  (NTD)  2:KVEAEVQIDRLITGRLQS(CH)  3:APHGVVFLHVTYVPAQEKNFTTAPAICHDGKAHFPREGVFVSNGTHWFVTQRNFYEPQIITTDNTF(CD) |
| 147 | 65.21 | 0.3100 | 1:TQDLFLPFFSNVTWFHAIHVSGTNGTKRFDNPVLPFNDGVYFASTEKSNIIRGWIFGTTLD(NTD)  2:KVEAEVQIDRLITGRLQS(CH)  3:APHGVVFLHVTYVPAQEKNFTTAPAICHDGKAHFPREGVFVSNGTHWFVTQRNFYEPQIITTDNTFVS(CD) |
| 148 | 65.75 | 0.3150 | 1:STQDLFLPFFSNVTWFHAIHVSGTNGTKRFDNPVLPFNDGVYFASTEKSNIIRGWIFGTTLD  (NTD)  2:KVEAEVQIDRLITGRLQS(CH)  3:APHGVVFLHVTYVPAQEKNFTTAPAICHDGKAHFPREGVFVSNGTHWFVTQRNFYEPQIITTDNTFVS(CD) |
| 149 | 66.22 | 0.3012 | 1:STQDLFLPFFSNVTWFHAIHVSGTNGTKRFDNPVLPFNDGVYFASTEKSNIIRGWIFGTTLD  (NTD)  2:KVEAEVQIDRLITGRLQS(CH)  3:APHGVVFLHVTYVPAQEKNFTTAPAICHDGKAHFPREGVFVSNGTHWFVTQRNFYEPQIITTDNTFVSG(CD) |
| 150 | 66.68 | 0.3034 | 1:STQDLFLPFFSNVTWFHAIHVSGTNGTKRFDNPVLPFNDGVYFASTEKSNIIRGWIFGTTLD  (NTD)  2:KVEAEVQIDRLITGRLQS(CH)  3:APHGVVFLHVTYVPAQEKNFTTAPAICHDGKAHFPREGVFVSNGTHWFVTQRNFYEPQIITTDNTFVSGN(CD) |
| 151 | 67.12 | 0.2994 | 1:STQDLFLPFFSNVTWFHAIHVSGTNGTKRFDNPVLPFNDGVYFASTEKSNIIRGWIFGTTLD  (NTD)  2:KVEAEVQIDRLITGRLQS(CH)  3:APHGVVFLHVTYVPAQEKNFTTAPAICHDGKAHFPREGVFVSNGTHWFVTQRNFYEPQIITTDNTFVSGNC(CD) |
| 152 | 67.48 | 0.2951 | 1:STQDLFLPFFSNVTWFHAIHVSGTNGTKRFDNPVLPFNDGVYFASTEKSNIIRGWIFGTTLD  (NTD)  2:KVEAEVQIDRLITGRLQS(CH)  3:APHGVVFLHVTYVPAQEKNFTTAPAICHDGKAHFPREGVFVSNGTHWFVTQRNFYEPQIITTDNTFVSGNCD(CD) |
| 153 | 67.91 | 0.3466 | 1:SAPHGVVFLHVTYVPAQEKNFTTAPAICHDGKAHFPREGVFVSNGTHWFVTQRNFYEPQIITTDNTFVSGNC(CD)  2:TEVPVAIHADQLTPTWRVYST  3:QDLFLPFFSNVTWFHAIHVSGTNGTKRFDNPVLPFNDGVYFASTEKSNIIRGWIFGTTLD(NTD) |
| 154 | 68.45 | 0.3545 | 1:QSAPHGVVFLHVTYVPAQEKNFTTAPAICHDGKAHFPREGVFVSNGTHWFVTQRNFYEPQIITTDNTFVSGNC(CD)  2:TEVPVAIHADQLTPTWRVYST  3:QDLFLPFFSNVTWFHAIHVSGTNGTKRFDNPVLPFNDGVYFASTEKSNIIRGWIFGTTLD(NTD) |
| 155 | 68.96 | 0.3422 | 1:QSAPHGVVFLHVTYVPAQEKNFTTAPAICHDGKAHFPREGVFVSNGTHWFVTQRNFYEPQIITTDNTFVSGNC(CD)  2:TEVPVAIHADQLTPTWRVYST  3:QDLFLPFFSNVTWFHAIHVSGTNGTKRFDNPVLPFNDGVYFASTEKSNIIRGWIFGTTLDS(NTD) |
| 156 | 69.41 | 0.3378 | 1:PQSAPHGVVFLHVTYVPAQEKNFTTAPAICHDGKAHFPREGVFVSNGTHWFVTQRNFYEPQIITTDNTFVSGNC(CD)  2:TEVPVAIHADQLTPTWRVYST  3:QDLFLPFFSNVTWFHAIHVSGTNGTKRFDNPVLPFNDGVYFASTEKSNIIRGWIFGTTLDS(NTD) |
| 157 | 69.83 | 0.3655 | 1:PQSAPHGVVFLHVTYVPAQEKNFTTAPAICHDGKAHFPREGVFVSNGTHWFVTQRNFYEPQIITTDNTFVSGNC(CD)  2:TEVPVAIHADQLTPTWRVYST  3:QDLFLPFFSNVTWFHAIHVSGTNGTKRFDNPVLPFNDGVYFASTEKSNIIRGWIFGTTLDSK  (NTD) |
| 158 | 70.2 | 0.3530 | 1:FPQSAPHGVVFLHVTYVPAQEKNFTTAPAICHDGKAHFPREGVFVSNGTHWFVTQRNFYEPQIITTDNTFVSGNC(CD)  2:TEVPVAIHADQLTPTWRVYST  3:QDLFLPFFSNVTWFHAIHVSGTNGTKRFDNPVLPFNDGVYFASTEKSNIIRGWIFGTTLDSK  (NTD) |
| 159 | 70.64 | 0.3299 | 1:QPTESIVRFPNITNLCPFGEVFNATRFAS  (RBD)  2:APHGVVFLHVTYVPAQEKNFTTAPAICHDGKAHFPREGVFVSNGTHWFVTQRNFYEPQIITTDNTFVS(CD)  3:TQDLFLPFFSNVTWFHAIHVSGTNGTKRFDNPVLPFNDGVYFASTEKSNIIRGWIFGTTLDS  (NTD) |
| 160 | 71.17 | 0.3433 | 1:STQDLFLPFFSNVTWFHAIHVSGTNGTKRFDNPVLPFNDGVYFASTEKSNIIRGWIFGTTLDS  (NTD)  2:APHGVVFLHVTYVPAQEKNFTTAPAICHDGKAHFPREGVFVSNGTHWFVTQRNFYEPQIITTDNTFV(CD)  3:QPTESIVRFPNITNLCPFGEVFNATRFASV  (RBD) |
| 161 | 71.69 | 0.3303 | 1:STQDLFLPFFSNVTWFHAIHVSGTNGTKRFDNPVLPFNDGVYFASTEKSNIIRGWIFGTTLDS  (NTD)  2:APHGVVFLHVTYVPAQEKNFTTAPAICHDGKAHFPREGVFVSNGTHWFVTQRNFYEPQIITTDNTFV(CD)  3:QPTESIVRFPNITNLCPFGEVFNATRFASVY  (RBD) |
| 162 | 72.16 | 0.3303 | 1:STQDLFLPFFSNVTWFHAIHVSGTNGTKRFDNPVLPFNDGVYFASTEKSNIIRGWIFGTTLDS  (NTD)  2:APHGVVFLHVTYVPAQEKNFTTAPAICHDGKAHFPREGVFVSNGTHWFVTQRNFYEPQIITTDNTFV(CD)  3:QPTESIVRFPNITNLCPFGEVFNATRFASVYA(RBD) |
| 163 | 72.58 | 0.3533 | 1:RVQPTESIVRFPNITNLCPFGEVFNATRFASVYA(RBD)  2:PHGVVFLHVTYVPAQEKNFTTAPAICHDGKAHFPREGVFVSNGTHWFVTQRNFYEPQIITTDNTFVS(CD)  3:TQDLFLPFFSNVTWFHAIHVSGTNGTKRFDNPVLPFNDGVYFASTEKSNIIRGWIFGTTLDS  (NTD) |
| 164 | 72.99 | 0.3797 | 1:RVQPTESIVRFPNITNLCPFGEVFNATRFASVYA(RBD)  2:PHGVVFLHVTYVPAQEKNFTTAPAICHDGKAHFPREGVFVSNGTHWFVTQRNFYEPQIITTDNTFVS(CD)  3:TQDLFLPFFSNVTWFHAIHVSGTNGTKRFDNPVLPFNDGVYFASTEKSNIIRGWIFGTTLDSK  (NTD) |
| 165 | 73.37 | 0.3798 | 1:NFRVQPTESIVRFPNITNLCPFGEVFNATRFASVYA(RBD)  2:PHGVVFLHVTYVPAQEKNFTTAPAICHDGKAHFPREGVFVSNGTHWFVTQRNFYEPQIITTDNTFVS(CD)  3:TQDLFLPFFSNVTWFHAIHVSGTNGTKRFDNPVLPFNDGVYFASTEKSNIIRGWIFGTTLDS  (NTD) |
| 166 | 73.91 | 0.3993 | 1:QSAPHGVVFLHVTYVPAQEKNFTTAPAICHDGKAHFPREGVFVSNGTHWFVTQRNFYEPQIITTDNTFVS(CD)  2:TQDLFLPFFSNVTWFHAIHVSGTNGTKRFDNPVLPFNDGVYFASTEKSNIIRGWIFGTTLDS  (NTD)  3:NFRVQPTESIVRFPNITNLCPFGEVFNATRFASV(RBD) |
| 167 | 74.43 | 0.3863 | 1:QSAPHGVVFLHVTYVPAQEKNFTTAPAICHDGKAHFPREGVFVSNGTHWFVTQRNFYEPQIITTDNTFVS(CD)  2:TQDLFLPFFSNVTWFHAIHVSGTNGTKRFDNPVLPFNDGVYFASTEKSNIIRGWIFGTTLDS  (NTD)  3:NFRVQPTESIVRFPNITNLCPFGEVFNATRFASVY(RBD) |
| 168 | 74.9 | 0.3860 | 1:QSAPHGVVFLHVTYVPAQEKNFTTAPAICHDGKAHFPREGVFVSNGTHWFVTQRNFYEPQIITTDNTFVS(CD)  2:TQDLFLPFFSNVTWFHAIHVSGTNGTKRFDNPVLPFNDGVYFASTEKSNIIRGWIFGTTLDS  (NTD)  3:NFRVQPTESIVRFPNITNLCPFGEVFNATRFASVYA(RBD) |
| 169 | 75.35 | 0.3817 | 1:PQSAPHGVVFLHVTYVPAQEKNFTTAPAICHDGKAHFPREGVFVSNGTHWFVTQRNFYEPQIITTDNTFVS(CD)  2:TQDLFLPFFSNVTWFHAIHVSGTNGTKRFDNPVLPFNDGVYFASTEKSNIIRGWIFGTTLDS  (NTD)  3:NFRVQPTESIVRFPNITNLCPFGEVFNATRFASVYA(RBD) |
| 170 | 75.75 | 0.3711 | 1:PQSAPHGVVFLHVTYVPAQEKNFTTAPAICHDGKAHFPREGVFVSNGTHWFVTQRNFYEPQIITTDNTFVS(CD)  2:TQDLFLPFFSNVTWFHAIHVSGTNGTKRFDNPVLPFNDGVYFASTEKSNIIRGWIFGTTLDS  (NTD)  3:NFRVQPTESIVRFPNITNLCPFGEVFNATRFASVYAW(RBD) |
| 171 | 76.12 | 0.3595 | 1:FPQSAPHGVVFLHVTYVPAQEKNFTTAPAICHDGKAHFPREGVFVSNGTHWFVTQRNFYEPQIITTDNTFVS(CD)  2:TQDLFLPFFSNVTWFHAIHVSGTNGTKRFDNPVLPFNDGVYFASTEKSNIIRGWIFGTTLDS  (NTD)  3:NFRVQPTESIVRFPNITNLCPFGEVFNATRFASVYAW(RBD) |
| 172 | 76.57 | 0.3616 | 1:STQDLFLPFFSNVTWFHAIHVSGTNGTKRFDNPVLPFNDGVYFASTEKSNIIRGWIFGTTLDS  (NTD)  2:APHGVVFLHVTYVPAQEKNFTTAPAICHDGKAHFPREGVFVSNGTHWFVTQRNFYEPQIITTDNTFVSG(CD)  3:IYQTSNFRVQPTESIVRFPNITNLCPFGEVFNATRFASVY(RBD) |
| 173 | 77.04 | 0.3614 | 1:STQDLFLPFFSNVTWFHAIHVSGTNGTKRFDNPVLPFNDGVYFASTEKSNIIRGWIFGTTLDS  (NTD)  2:APHGVVFLHVTYVPAQEKNFTTAPAICHDGKAHFPREGVFVSNGTHWFVTQRNFYEPQIITTDNTFVSG(CD)  3:IYQTSNFRVQPTESIVRFPNITNLCPFGEVFNATRFASVYA(RBD) |
| 174 | 77.44 | 0.3512 | 1:STQDLFLPFFSNVTWFHAIHVSGTNGTKRFDNPVLPFNDGVYFASTEKSNIIRGWIFGTTLDS  (NTD)  2:APHGVVFLHVTYVPAQEKNFTTAPAICHDGKAHFPREGVFVSNGTHWFVTQRNFYEPQIITTDNTFVSG(CD)  3:IYQTSNFRVQPTESIVRFPNITNLCPFGEVFNATRFASVYAW(RBD) |
| 175 | 77.85 | 0.4263 | 1:QEKNFTTAPAICHDGKAHFPREGVFVSNGTHWFVTQRNFYEPQIITTDNTFVSGNC(CD)  2:TEVPVAIHADQLTPTWRVYSTGSNVFQTRAGCLIGAEHVNNSYECDIPIGAGICAS  3:TQDLFLPFFSNVTWFHAIHVSGTNGTKRFDNPVLPFNDGVYFASTEKSNIIRGWIFGTTLDSK  (NTD) |
| 176 | 78.37 | 0.3765 | 1:SAPHGVVFLHVTYVPAQEKNFTTAPAICHDGKAHFPREGVFVSNGTHWFVTQRNFYEPQIITTDNTFVS(CD)  2:TQDLFLPFFSNVTWFHAIHVSGTNGTKRFDNPVLPFNDGVYFASTEKSNIIRGWIFGTTL(NTD)  3:KSFTVEKGIYQTSNFRVQPTESIVRFPNITNLCPFGEVFNATRFASV(NTD,RBD) |
| 177 | 78.92 | 0.3831 | 1:QSAPHGVVFLHVTYVPAQEKNFTTAPAICHDGKAHFPREGVFVSNGTHWFVTQRNFYEPQIITTDNTFVS(CD)  2:TQDLFLPFFSNVTWFHAIHVSGTNGTKRFDNPVLPFNDGVYFASTEKSNIIRGWIFGTTL(NTD)  3:KSFTVEKGIYQTSNFRVQPTESIVRFPNITNLCPFGEVFNATRFASV(NTD,RBD) |
| 178 | 79.44 | 0.3711 | 1:QSAPHGVVFLHVTYVPAQEKNFTTAPAICHDGKAHFPREGVFVSNGTHWFVTQRNFYEPQIITTDNTFVS(CD)  2:TQDLFLPFFSNVTWFHAIHVSGTNGTKRFDNPVLPFNDGVYFASTEKSNIIRGWIFGTTL(NTD)  3:KSFTVEKGIYQTSNFRVQPTESIVRFPNITNLCPFGEVFNATRFASVY(NTD,RBD) |
| 179 | 79.9 | 0.3709 | 1:QSAPHGVVFLHVTYVPAQEKNFTTAPAICHDGKAHFPREGVFVSNGTHWFVTQRNFYEPQIITTDNTFVS(CD)  2:TQDLFLPFFSNVTWFHAIHVSGTNGTKRFDNPVLPFNDGVYFASTEKSNIIRGWIFGTTL(NTD)  3:KSFTVEKGIYQTSNFRVQPTESIVRFPNITNLCPFGEVFNATRFASVYA(NTD,RBD) |
| 180 | 80.36 | 0.3669 | 1:PQSAPHGVVFLHVTYVPAQEKNFTTAPAICHDGKAHFPREGVFVSNGTHWFVTQRNFYEPQIITTDNTFVS(CD)  2:TQDLFLPFFSNVTWFHAIHVSGTNGTKRFDNPVLPFNDGVYFASTEKSNIIRGWIFGTTL(NTD)  3:KSFTVEKGIYQTSNFRVQPTESIVRFPNITNLCPFGEVFNATRFASVYA(NTD,RBD) |
| 181 | 80.76 | 0.3571 | 1:PQSAPHGVVFLHVTYVPAQEKNFTTAPAICHDGKAHFPREGVFVSNGTHWFVTQRNFYEPQIITTDNTFVS(CD)  2:TQDLFLPFFSNVTWFHAIHVSGTNGTKRFDNPVLPFNDGVYFASTEKSNIIRGWIFGTTL(NTD)  3:KSFTVEKGIYQTSNFRVQPTESIVRFPNITNLCPFGEVFNATRFASVYAW(NTD,RBD) |
| 182 | 81.13 | 0.3462 | 1:FPQSAPHGVVFLHVTYVPAQEKNFTTAPAICHDGKAHFPREGVFVSNGTHWFVTQRNFYEPQIITTDNTFVS(CD)  2:TQDLFLPFFSNVTWFHAIHVSGTNGTKRFDNPVLPFNDGVYFASTEKSNIIRGWIFGTTL(NTD)  3:KSFTVEKGIYQTSNFRVQPTESIVRFPNITNLCPFGEVFNATRFASVYAW(NTD,RBD) |
| 183 | 81.4 | 0.3463 | 1:SFPQSAPHGVVFLHVTYVPAQEKNFTTAPAICHDGKAHFPREGVFVSNGTHWFVTQRNFYEPQIITTDNTFVS(CD)  2:TQDLFLPFFSNVTWFHAIHVSGTNGTKRFDNPVLPFNDGVYFASTEKSNIIRGWIFGTTL(NTD)  3:KSFTVEKGIYQTSNFRVQPTESIVRFPNITNLCPFGEVFNATRFASVYAW(NTD,RBD) |
| 184 | 81.87 | 0.4447 | 1:LPFFSNVTWFHAIHVSGTNGTKRFDNPVLPFNDGVYFASTEKSNIIRGWIFGTTLDS(NTD)  2:APHGVVFLHVTYVPAQEKNFTTAPAICHDGKAHFPREGVFVSNGTHWFVTQRNFYEPQIITTDNTFVSGNC(CD)  3:TEVPVAIHADQLTPTWRVYSTGSNVFQTRAGCLIGAEHVNNSYECDIPIGAGICAS |
| 185 | 82.34 | 0.4417 | 1:FLPFFSNVTWFHAIHVSGTNGTKRFDNPVLPFNDGVYFASTEKSNIIRGWIFGTTLDS(NTD)  2:APHGVVFLHVTYVPAQEKNFTTAPAICHDGKAHFPREGVFVSNGTHWFVTQRNFYEPQIITTDNTFVSGNC(CD)  3:TEVPVAIHADQLTPTWRVYSTGSNVFQTRAGCLIGAEHVNNSYECDIPIGAGICAS |
| 186 | 82.81 | 0.4483 | 1:FLPFFSNVTWFHAIHVSGTNGTKRFDNPVLPFNDGVYFASTEKSNIIRGWIFGTTLDS(NTD)  2:APHGVVFLHVTYVPAQEKNFTTAPAICHDGKAHFPREGVFVSNGTHWFVTQRNFYEPQIITTDNTFVSGNC(CD)  3:TEVPVAIHADQLTPTWRVYSTGSNVFQTRAGCLIGAEHVNNSYECDIPIGAGICASY |
| 187 | 83.34 | 0.4302 | 1:QDLFLPFFSNVTWFHAIHVSGTNGTKRFDNPVLPFNDGVYFASTEKSNIIRGWIFGTTLDS(NTD)  2:APHGVVFLHVTYVPAQEKNFTTAPAICHDGKAHFPREGVFVSNGTHWFVTQRNFYEPQIITTDNTFVSGNC(CD)  3:TEVPVAIHADQLTPTWRVYSTGSNVFQTRAGCLIGAEHVNNSYECDIPIGAGICA |
| 188 | 83.88 | 0.4373 | 1:APHGVVFLHVTYVPAQEKNFTTAPAICHDGKAHFPREGVFVSNGTHWFVTQRNFYEPQIITTDNTFVSGNC(CD)  2:TEVPVAIHADQLTPTWRVYSTGSNVFQTRAGCLIGAEHVNNSYECDIPIGAGICAS  3:TQDLFLPFFSNVTWFHAIHVSGTNGTKRFDNPVLPFNDGVYFASTEKSNIIRGWIFGTTLD(NTD) |
| 189 | 84.43 | 0.4332 | 1:SAPHGVVFLHVTYVPAQEKNFTTAPAICHDGKAHFPREGVFVSNGTHWFVTQRNFYEPQIITTDNTFVSGNC(CD)  2:TEVPVAIHADQLTPTWRVYSTGSNVFQTRAGCLIGAEHVNNSYECDIPIGAGICAS  3:TQDLFLPFFSNVTWFHAIHVSGTNGTKRFDNPVLPFNDGVYFASTEKSNIIRGWIFGTTLD(NTD) |
| 190 | 84.97 | 0.4391 | 1:QSAPHGVVFLHVTYVPAQEKNFTTAPAICHDGKAHFPREGVFVSNGTHWFVTQRNFYEPQIITTDNTFVSGNC(CD)  2:TEVPVAIHADQLTPTWRVYSTGSNVFQTRAGCLIGAEHVNNSYECDIPIGAGICAS  3:TQDLFLPFFSNVTWFHAIHVSGTNGTKRFDNPVLPFNDGVYFASTEKSNIIRGWIFGTTLD(NTD) |
| 191 | 85.48 | 0.4288 | 1:QSAPHGVVFLHVTYVPAQEKNFTTAPAICHDGKAHFPREGVFVSNGTHWFVTQRNFYEPQIITTDNTFVSGNC(CD)  2:TEVPVAIHADQLTPTWRVYSTGSNVFQTRAGCLIGAEHVNNSYECDIPIGAGICAS  3:TQDLFLPFFSNVTWFHAIHVSGTNGTKRFDNPVLPFNDGVYFASTEKSNIIRGWIFGTTLDS  (NTD) |
| 192 | 85.94 | 0.4247 | 1:PQSAPHGVVFLHVTYVPAQEKNFTTAPAICHDGKAHFPREGVFVSNGTHWFVTQRNFYEPQIITTDNTFVSGNC(CD)  2:TEVPVAIHADQLTPTWRVYSTGSNVFQTRAGCLIGAEHVNNSYECDIPIGAGICAS  3:TQDLFLPFFSNVTWFHAIHVSGTNGTKRFDNPVLPFNDGVYFASTEKSNIIRGWIFGTTLDS  (NTD) |
| 193 | 86.4 | 0.4573 | 1:QSAPHGVVFLHVTYVPAQEKNFTTAPAICHDGKAHFPREGVFVSNGTHWFVTQRNFYEPQIITTDNTFVSGNC(CD)  2:TEVPVAIHADQLTPTWRVYSTGSNVFQTRAGCLIGAEHVNNSYECDIPIGAGICASYQTQ  3:DLFLPFFSNVTWFHAIHVSGTNGTKRFDNPVLPFNDGVYFASTEKSNIIRGWIFGTTLDS(NTD) |
| 194 | 86.85 | 0.4532 | 1:PQSAPHGVVFLHVTYVPAQEKNFTTAPAICHDGKAHFPREGVFVSNGTHWFVTQRNFYEPQIITTDNTFVSGNC(CD)  2:TEVPVAIHADQLTPTWRVYSTGSNVFQTRAGCLIGAEHVNNSYECDIPIGAGICASYQTQ  3:DLFLPFFSNVTWFHAIHVSGTNGTKRFDNPVLPFNDGVYFASTEKSNIIRGWIFGTTLDS(NTD) |
| 195 | 87.27 | 0.4748 | 1:PQSAPHGVVFLHVTYVPAQEKNFTTAPAICHDGKAHFPREGVFVSNGTHWFVTQRNFYEPQIITTDNTFVSGNC(CD)  2:TEVPVAIHADQLTPTWRVYSTGSNVFQTRAGCLIGAEHVNNSYECDIPIGAGICASYQTQ  3:DLFLPFFSNVTWFHAIHVSGTNGTKRFDNPVLPFNDGVYFASTEKSNIIRGWIFGTTLDSK(NTD) |
| 196 | 87.64 | 0.4642 | 1:FPQSAPHGVVFLHVTYVPAQEKNFTTAPAICHDGKAHFPREGVFVSNGTHWFVTQRNFYEPQIITTDNTFVSGNC(CD)  2:TEVPVAIHADQLTPTWRVYSTGSNVFQTRAGCLIGAEHVNNSYECDIPIGAGICASYQTQ  3:DLFLPFFSNVTWFHAIHVSGTNGTKRFDNPVLPFNDGVYFASTEKSNIIRGWIFGTTLDSK(NTD) |
| 197 | 87.92 | 0.4582 | 1:FPQSAPHGVVFLHVTYVPAQEKNFTTAPAICHDGKAHFPREGVFVSNGTHWFVTQRNFYEPQIITTDNTFVSGNC(CD)  2:TEVPVAIHADQLTPTWRVYSTGSNVFQTRAGCLIGAEHVNNSYECDIPIGAGICASYQTQ  3:DLFLPFFSNVTWFHAIHVSGTNGTKRFDNPVLPFNDGVYFASTEKSNIIRGWIFGTTLDSKT  (NTD) |
| 198 | 88.19 | 0.4577 | 1:SFPQSAPHGVVFLHVTYVPAQEKNFTTAPAICHDGKAHFPREGVFVSNGTHWFVTQRNFYEPQIITTDNTFVSGNC(CD)  2:TEVPVAIHADQLTPTWRVYSTGSNVFQTRAGCLIGAEHVNNSYECDIPIGAGICASYQTQ  3:DLFLPFFSNVTWFHAIHVSGTNGTKRFDNPVLPFNDGVYFASTEKSNIIRGWIFGTTLDSKT  (NTD) |
| 199 | 88.49 | 0.4588 | 1:LMSFPQSAPHGVVFLHVTYVPAQEKNFTTAPAICHDGKAHFPREGVFVSNGTHWFVTQRNFYEPQIITTDNTFVSGNC(CD)  2:TEVPVAIHADQLTPTWRVYSTGSNVFQTRAGCLIGAEHVNNSYECDIPIGAGICASYQTQ  3:DLFLPFFSNVTWFHAIHVSGTNGTKRFDNPVLPFNDGVYFASTEKSNIIRGWIFGTTLDSK(NTD) |
| 200 | 88.86 | 0.4584 | 1:HLMSFPQSAPHGVVFLHVTYVPAQEKNFTTAPAICHDGKAHFPREGVFVSNGTHWFVTQRNFYEPQIITTDNTFVSGNC(CD)  2:TEVPVAIHADQLTPTWRVYSTGSNVFQTRAGCLIGAEHVNNSYECDIPIGAGICASYQTQ  3:DLFLPFFSNVTWFHAIHVSGTNGTKRFDNPVLPFNDGVYFASTEKSNIIRGWIFGTTLDSK(NTD) |
| 201 | 89.14 | 0.4526 | 1:HLMSFPQSAPHGVVFLHVTYVPAQEKNFTTAPAICHDGKAHFPREGVFVSNGTHWFVTQRNFYEPQIITTDNTFVSGNC(CD)  2:TEVPVAIHADQLTPTWRVYSTGSNVFQTRAGCLIGAEHVNNSYECDIPIGAGICASYQTQ  3:DLFLPFFSNVTWFHAIHVSGTNGTKRFDNPVLPFNDGVYFASTEKSNIIRGWIFGTTLDSKT  (NTD) |
| 202 | 89.38 | 0.4584 | 1:HLMSFPQSAPHGVVFLHVTYVPAQEKNFTTAPAICHDGKAHFPREGVFVSNGTHWFVTQRNFYEPQIITTDNTFVSGNC(CD)  2:TEVPVAIHADQLTPTWRVYSTGSNVFQTRAGCLIGAEHVNNSYECDIPIGAGICASYQTQ  3:DLFLPFFSNVTWFHAIHVSGTNGTKRFDNPVLPFNDGVYFASTEKSNIIRGWIFGTTLDSKTQ  (NTD) |
| 203 | 89.75 | 0.4387 | 1:STQDLFLPFFSNVTWFHAIHVSGTNGTKRFDNPVLPFNDGVYFASTEKSNIIRGWIFGTT(NTD)  2:APAICHDGKAHFPREGVFVSNGTHWFVTQRNFYEPQIITTDNTFVSGNC(CD)  3:TEVPVAIHADQLTPTWRVYSTGSNVFQTRAGCLIGAEHVNNSYECDIPIGAGICASYQT  4:SNFRVQPTESIVRFPNITNLCPFGEVFNATRFASV(RBD) |
| 204 | 90.27 | 0.4279 | 1:STQDLFLPFFSNVTWFHAIHVSGTNGTKRFDNPVLPFNDGVYFASTEKSNIIRGWIFGTT(NTD)  2:APAICHDGKAHFPREGVFVSNGTHWFVTQRNFYEPQIITTDNTFVSGNC(CD)  3:TEVPVAIHADQLTPTWRVYSTGSNVFQTRAGCLIGAEHVNNSYECDIPIGAGICASYQT  4:SNFRVQPTESIVRFPNITNLCPFGEVFNATRFASVY(RBD) |
| 205 | 90.74 | 0.4275 | 1:STQDLFLPFFSNVTWFHAIHVSGTNGTKRFDNPVLPFNDGVYFASTEKSNIIRGWIFGTT(NTD)  2:APAICHDGKAHFPREGVFVSNGTHWFVTQRNFYEPQIITTDNTFVSGNC(CD)  3:TEVPVAIHADQLTPTWRVYSTGSNVFQTRAGCLIGAEHVNNSYECDIPIGAGICASYQT  4:SNFRVQPTESIVRFPNITNLCPFGEVFNATRFASVYA(RBD) |
| 206 | 91.14 | 0.4186 | 1:STQDLFLPFFSNVTWFHAIHVSGTNGTKRFDNPVLPFNDGVYFASTEKSNIIRGWIFGTT(NTD)  2:APAICHDGKAHFPREGVFVSNGTHWFVTQRNFYEPQIITTDNTFVSGNC(CD)  3:TEVPVAIHADQLTPTWRVYSTGSNVFQTRAGCLIGAEHVNNSYECDIPIGAGICASYQT  4:SNFRVQPTESIVRFPNITNLCPFGEVFNATRFASVYAW(RBD) |
| 207 | 91.58 | 0.4378 | 1:STQDLFLPFFSNVTWFHAIHVSGTNGTKRFDNPVLPFNDGVYFASTEKSNIIRGWIFGTTLDS  (NTD)  2:APHGVVFLHVTYVPAQEKNFTTAPAICHDGKAHFPREGVFVSNGTHWFVTQRNFYEPQIITTDNTF(CD)  3:QQFGRDIADTTDAVRDPQTLEILDITPCSFGGVSVITPGTNTSN  4:FRVQPTESIVRFPNITNLCPFGEVFNATRFASVY(RBD) |
| 208 | 92.05 | 0.4435 | 1:FQQFGRDIADTTDAVRDPQTLEILDITPCSFGGVSVITPGTNTSN  2:FRVQPTESIVRFPNITNLCPFGEVFNATRFASVYA(RBD)  3:PHGVVFLHVTYVPAQEKNFTTAPAICHDGKAHFPREGVFVSNGTHWFVTQRNFYEPQIITTDNTFVS(CD)  4:TQDLFLPFFSNVTWFHAIHVSGTNGTKRFDNPVLPFNDGVYFASTEKSNIIRGWIFGTTLD(NTD) |
| 209 | 92.58 | 0.4259 | 1:EKNFTTAPAICHDGKAHFPREGVFVSNGTHWFVTQRNFYEPQIITTDNTFVSGNC(CD)  2:TEVPVAIHADQLTPTWRVYSTGSNVFQTRAGCLIGAEHVNNSYECDIPIGAGICASYQT  3:SNFRVQPTESIVRFPNITNLCPFGEVFNATRFAS(RBD)  4:TQDLFLPFFSNVTWFHAIHVSGTNGTKRFDNPVLPFNDGVYFASTEKSNIIRGWIFGTTLD(NTD) |
| 210 | 93.09 | 0.4166 | 1:EKNFTTAPAICHDGKAHFPREGVFVSNGTHWFVTQRNFYEPQIITTDNTFVSGNC(CD)  2:TEVPVAIHADQLTPTWRVYSTGSNVFQTRAGCLIGAEHVNNSYECDIPIGAGICASYQT  3:SNFRVQPTESIVRFPNITNLCPFGEVFNATRFAS(RBD)  4:TQDLFLPFFSNVTWFHAIHVSGTNGTKRFDNPVLPFNDGVYFASTEKSNIIRGWIFGTTLDS  (NTD) |
| 211 | 93.59 | 0.4269 | 1:QEKNFTTAPAICHDGKAHFPREGVFVSNGTHWFVTQRNFYEPQIITTDNTFVSGNC(CD)  2:TEVPVAIHADQLTPTWRVYSTGSNVFQTRAGCLIGAEHVNNSYECDIPIGAGICASYQT  3:SNFRVQPTESIVRFPNITNLCPFGEVFNATRFAS(RBD)  4:TQDLFLPFFSNVTWFHAIHVSGTNGTKRFDNPVLPFNDGVYFASTEKSNIIRGWIFGTTLDS  (NTD) |
| 212 | 94.05 | 0.4388 | 1:STQDLFLPFFSNVTWFHAIHVSGTNGTKRFDNPVLPFNDGVYFASTEKSNIIRGWIFGTTLDSK  (NTD)  2:NFTTAPAICHDGKAHFPREGVFVSNGTHWFVTQRNFYEPQIITTDNTFVSGNC(CD)  3:TEVPVAIHADQLTPTWRVYSTGSNVFQTRAGCLIGAEHVNNSYECDIPIGAGICASYQT  4:SNFRVQPTESIVRFPNITNLCPFGEVFNATRFASVY(RBD) |
| 213 | 94.52 | 0.4383 | 1:STQDLFLPFFSNVTWFHAIHVSGTNGTKRFDNPVLPFNDGVYFASTEKSNIIRGWIFGTTLDSK  (NTD)  2:NFTTAPAICHDGKAHFPREGVFVSNGTHWFVTQRNFYEPQIITTDNTFVSGNC(CD)  3:TEVPVAIHADQLTPTWRVYSTGSNVFQTRAGCLIGAEHVNNSYECDIPIGAGICASYQT  4:SNFRVQPTESIVRFPNITNLCPFGEVFNATRFASVYA(RBD) |
| 214 | 94.92 | 0.4297 | 1:STQDLFLPFFSNVTWFHAIHVSGTNGTKRFDNPVLPFNDGVYFASTEKSNIIRGWIFGTTLDSK  (NTD)  2:NFTTAPAICHDGKAHFPREGVFVSNGTHWFVTQRNFYEPQIITTDNTFVSGNC(CD)  3:TEVPVAIHADQLTPTWRVYSTGSNVFQTRAGCLIGAEHVNNSYECDIPIGAGICASYQT  4:SNFRVQPTESIVRFPNITNLCPFGEVFNATRFASVYAW(RBD) |
| 215 | 95.29 | 0.4693 | 1:QSAPHGVVFLHVTYVPAQEKNFTTAPAICHDGKAHFPREGVFVSNGTHWFVTQRNFYEPQIITTDNTFVS(CD)  2:TQDLFLPFFSNVTWFHAIHVSGTNGTKRFDNPVLPFNDGVYFASTEKSNIIRGWIFGTTLDSK  (NTD)  3:FLPFQQFGRDIADTTDAVRDPQTLEILDITPCSFGGVSVITPGTNTSN  4:FRVQPTESIVRFPNITNLCPFGEVFNATRFASVY(RBD) |
| 216 | 95.76 | 0.4687 | 1:QSAPHGVVFLHVTYVPAQEKNFTTAPAICHDGKAHFPREGVFVSNGTHWFVTQRNFYEPQIITTDNTFVS(CD)  2:TQDLFLPFFSNVTWFHAIHVSGTNGTKRFDNPVLPFNDGVYFASTEKSNIIRGWIFGTTLDSK  (NTD)  3:FLPFQQFGRDIADTTDAVRDPQTLEILDITPCSFGGVSVITPGTNTSN  4:FRVQPTESIVRFPNITNLCPFGEVFNATRFASVYA(RBD) |
| 217 | 96.21 | 0.4650 | 1:PQSAPHGVVFLHVTYVPAQEKNFTTAPAICHDGKAHFPREGVFVSNGTHWFVTQRNFYEPQIITTDNTFVS(CD)  2:TQDLFLPFFSNVTWFHAIHVSGTNGTKRFDNPVLPFNDGVYFASTEKSNIIRGWIFGTTLDSK  (NTD)  3:FLPFQQFGRDIADTTDAVRDPQTLEILDITPCSFGGVSVITPGTNTSN  4:FRVQPTESIVRFPNITNLCPFGEVFNATRFASVYA(RBD) |
| 218 | 96.61 | 0.4564 | 1:PQSAPHGVVFLHVTYVPAQEKNFTTAPAICHDGKAHFPREGVFVSNGTHWFVTQRNFYEPQIITTDNTFVS(CD)  2:TQDLFLPFFSNVTWFHAIHVSGTNGTKRFDNPVLPFNDGVYFASTEKSNIIRGWIFGTTLDSK  (NTD)  3:FLPFQQFGRDIADTTDAVRDPQTLEILDITPCSFGGVSVITPGTNTSN  4:FRVQPTESIVRFPNITNLCPFGEVFNATRFASVYAW(RBD) |
| 219 | 97.02 | 0.4554 | 1:FFSNVTWFHAIHVSGTNGTKRFDNPVLPFNDGVYFASTEKSNIIRGWIFGTTLDS(NTD)  2:APHGVVFLHVTYVPAQEKNFTTAPAICHDGKAHFPREGVFVSNGTHWFVTQRNFYEPQIITTDNTFVSGNC(CD)  3:TEVPVAIHADQLTPTWRVYSTGSNVFQTRAGCLIGAEHVNNSYECDIPIGAGICASYQT  4:SNFRVQPTESIVRFPNITNLCPFGEVFNATRFAS(RBD) |
| 220 | 97.59 | 0.4639 | 1:FFSNVTWFHAIHVSGTNGTKRFDNPVLPFNDGVYFASTEKSNIIRGWIFGTTLDS(NTD)  2:APHGVVFLHVTYVPAQEKNFTTAPAICHDGKAHFPREGVFVSNGTHWFVTQRNFYEPQIITTDNTFVSGNC(CD)  3:TEVPVAIHADQLTPTWRVYSTGSNVFQTRAGCLIGAEHVNNSYECDIPIGAGICASYQT  4:SNFRVQPTESIVRFPNITNLCPFGEVFNATRFASV(RBD) |
| 221 | 98.12 | 0.4539 | 1:FFSNVTWFHAIHVSGTNGTKRFDNPVLPFNDGVYFASTEKSNIIRGWIFGTTLDS(NTD)  2:APHGVVFLHVTYVPAQEKNFTTAPAICHDGKAHFPREGVFVSNGTHWFVTQRNFYEPQIITTDNTFVSGNC(CD)  3:TEVPVAIHADQLTPTWRVYSTGSNVFQTRAGCLIGAEHVNNSYECDIPIGAGICASYQT  4:SNFRVQPTESIVRFPNITNLCPFGEVFNATRFASVY(RBD) |
| 222 | 98.6 | 0.4610 | 1:PFFSNVTWFHAIHVSGTNGTKRFDNPVLPFNDGVYFASTEKSNIIRGWIFGTTLDS(NTD)  2:APHGVVFLHVTYVPAQEKNFTTAPAICHDGKAHFPREGVFVSNGTHWFVTQRNFYEPQIITTDNTFVSGNC(CD)  1:PFFSNVTWFHAIHVSGTNGTKRFDNPVLPFNDGVYFASTEKSNIIRGWIFGTTLDS(NTD)  2:APHGVVFLHVTYVPAQEKNFTTAPAICHDGKAHFPREGVFVSNGTHWFVTQRNFYEPQIITTDNTFVSGNC(CD)  3:TEVPVAIHADQLTPTWRVYSTGSNVFQTRAGCLIGAEHVNNSYECDIPIGAGICASYQT  4: SNFRVQPTESIVRFPNITNLCPFGEVFNATRFASVY(RBD) |
| 223 | 99.12 | 0.4624 | 1:LPFFSNVTWFHAIHVSGTNGTKRFDNPVLPFNDGVYFASTEKSNIIRGWIFGTTLDS(NTD)  2:APHGVVFLHVTYVPAQEKNFTTAPAICHDGKAHFPREGVFVSNGTHWFVTQRNFYEPQIITTDNTFVSGNC(CD)  3:TEVPVAIHADQLTPTWRVYSTGSNVFQTRAGCLIGAEHVNNSYECDIPIGAGICASYQT  4:SNFRVQPTESIVRFPNITNLCPFGEVFNATRFASVY(RBD) |
| 224 | 99.59 | 0.4598 | 1:FLPFFSNVTWFHAIHVSGTNGTKRFDNPVLPFNDGVYFASTEKSNIIRGWIFGTTLDS(NTD)  2:APHGVVFLHVTYVPAQEKNFTTAPAICHDGKAHFPREGVFVSNGTHWFVTQRNFYEPQIITTDNTFVSGNC(CD)  3:TEVPVAIHADQLTPTWRVYSTGSNVFQTRAGCLIGAEHVNNSYECDIPIGAGICASYQT  4:SNFRVQPTESIVRFPNITNLCPFGEVFNATRFASVY(RBD) |
| 225 | 100.06 | 0.4593 | 1:FLPFFSNVTWFHAIHVSGTNGTKRFDNPVLPFNDGVYFASTEKSNIIRGWIFGTTLDS(NTD)  2:APHGVVFLHVTYVPAQEKNFTTAPAICHDGKAHFPREGVFVSNGTHWFVTQRNFYEPQIITTDNTFVSGNC(CD)  3:TEVPVAIHADQLTPTWRVYSTGSNVFQTRAGCLIGAEHVNNSYECDIPIGAGICASYQT  4:SNFRVQPTESIVRFPNITNLCPFGEVFNATRFASVYA(RBD) |
| 226 | 100.61 | 0.4585 | 1:QDLFLPFFSNVTWFHAIHVSGTNGTKRFDNPVLPFNDGVYFASTEKSNIIRGWIFGTTLDS(NTD)  2:APHGVVFLHVTYVPAQEKNFTTAPAICHDGKAHFPREGVFVSNGTHWFVTQRNFYEPQIITTDNTFVSGNC(CD)  3:TEVPVAIHADQLTPTWRVYSTGSNVFQTRAGCLIGAEHVNNSYECDIPIGAGICASYQT  4:SNFRVQPTESIVRFPNITNLCPFGEVFNATRFASV(RBD) |
| 227 | 101.14 | 0.4594 | 1:TQDLFLPFFSNVTWFHAIHVSGTNGTKRFDNPVLPFNDGVYFASTEKSNIIRGWIFGTTLDS  (NTD)  2:APHGVVFLHVTYVPAQEKNFTTAPAICHDGKAHFPREGVFVSNGTHWFVTQRNFYEPQIITTDNTFVSGNC(CD)  3:TEVPVAIHADQLTPTWRVYSTGSNVFQTRAGCLIGAEHVNNSYECDIPIGAGICASYQT  4:SNFRVQPTESIVRFPNITNLCPFGEVFNATRFASV(RBD) |
| 228 | 101.67 | 0.4620 | 1:STQDLFLPFFSNVTWFHAIHVSGTNGTKRFDNPVLPFNDGVYFASTEKSNIIRGWIFGTTLDS  (NTD)  2:APHGVVFLHVTYVPAQEKNFTTAPAICHDGKAHFPREGVFVSNGTHWFVTQRNFYEPQIITTDNTFVSGNC(CD)  3:TEVPVAIHADQLTPTWRVYSTGSNVFQTRAGCLIGAEHVNNSYECDIPIGAGICASYQT  4:SNFRVQPTESIVRFPNITNLCPFGEVFNATRFASV(RBD) |
| 229 | 102.19 | 0.4523 | 1:STQDLFLPFFSNVTWFHAIHVSGTNGTKRFDNPVLPFNDGVYFASTEKSNIIRGWIFGTTLDS  (NTD)  2:APHGVVFLHVTYVPAQEKNFTTAPAICHDGKAHFPREGVFVSNGTHWFVTQRNFYEPQIITTDNTFVSGNC(CD)  3:TEVPVAIHADQLTPTWRVYSTGSNVFQTRAGCLIGAEHVNNSYECDIPIGAGICASYQT  4:SNFRVQPTESIVRFPNITNLCPFGEVFNATRFASVY(RBD) |
| 230 | 102.66 | 0.4518 | 1:STQDLFLPFFSNVTWFHAIHVSGTNGTKRFDNPVLPFNDGVYFASTEKSNIIRGWIFGTTLDS  (NTD)  2:APHGVVFLHVTYVPAQEKNFTTAPAICHDGKAHFPREGVFVSNGTHWFVTQRNFYEPQIITTDNTFVSGNC(CD)  3:TEVPVAIHADQLTPTWRVYSTGSNVFQTRAGCLIGAEHVNNSYECDIPIGAGICASYQT  4:SNFRVQPTESIVRFPNITNLCPFGEVFNATRFASVYA(RBD) |
| 231 | 103.06 | 0.4438 | 1:STQDLFLPFFSNVTWFHAIHVSGTNGTKRFDNPVLPFNDGVYFASTEKSNIIRGWIFGTTLDS  (NTD)  2:APHGVVFLHVTYVPAQEKNFTTAPAICHDGKAHFPREGVFVSNGTHWFVTQRNFYEPQIITTDNTFVSGNC(CD)  3:TEVPVAIHADQLTPTWRVYSTGSNVFQTRAGCLIGAEHVNNSYECDIPIGAGICASYQT  4:SNFRVQPTESIVRFPNITNLCPFGEVFNATRFASVYAW(RBD) |
| 232 | 103.32 | 0.4845 | 1:STQDLFLPFFSNVTWFHAIHVSGTNGTKRFDNPVLPFNDGVYFASTEKSNIIRGWIFGTTLDSKTQS(NTD)  2:APHGVVFLHVTYVPAQEKNFTTAPAICHDGKAHFPREGVFVSNGTHWFVTQRNFYEPQIITTDNTFVSGNC(CD)  3:TEVPVAIHADQLTPTWRVYSTGSNVFQTRAGCLIGAEHVNNSYECDIPIGAGICASYQT  4:SNFRVQPTESIVRFPNITNLCPFGEVFNATRFASV(RBD) |
| 233 | 103.85 | 0.4749 | 1:STQDLFLPFFSNVTWFHAIHVSGTNGTKRFDNPVLPFNDGVYFASTEKSNIIRGWIFGTTLDSKTQS(NTD)  2:APHGVVFLHVTYVPAQEKNFTTAPAICHDGKAHFPREGVFVSNGTHWFVTQRNFYEPQIITTDNTFVSGNC(CD)  3:TEVPVAIHADQLTPTWRVYSTGSNVFQTRAGCLIGAEHVNNSYECDIPIGAGICASYQT  4:SNFRVQPTESIVRFPNITNLCPFGEVFNATRFASVY(RBD) |
| 234 | 104.31 | 0.4743 | 1:STQDLFLPFFSNVTWFHAIHVSGTNGTKRFDNPVLPFNDGVYFASTEKSNIIRGWIFGTTLDSKTQS(NTD)  2:APHGVVFLHVTYVPAQEKNFTTAPAICHDGKAHFPREGVFVSNGTHWFVTQRNFYEPQIITTDNTFVSGNC(CD)  3:TEVPVAIHADQLTPTWRVYSTGSNVFQTRAGCLIGAEHVNNSYECDIPIGAGICASYQT  4:SNFRVQPTESIVRFPNITNLCPFGEVFNATRFASVYA(RBD) |
| 235 | 104.71 | 0.4663 | 1:STQDLFLPFFSNVTWFHAIHVSGTNGTKRFDNPVLPFNDGVYFASTEKSNIIRGWIFGTTLDSKTQS(NTD)  2:APHGVVFLHVTYVPAQEKNFTTAPAICHDGKAHFPREGVFVSNGTHWFVTQRNFYEPQIITTDNTFVSGNC(CD)  3:TEVPVAIHADQLTPTWRVYSTGSNVFQTRAGCLIGAEHVNNSYECDIPIGAGICASYQT  4:SNFRVQPTESIVRFPNITNLCPFGEVFNATRFASVYAW(RBD) |
| 236 | 104.84 | 0.4399 | 1:STQDLFLPFFSNVTWFHAIHVSGTNGTKRFDNPVLPFNDGVYFASTEKSNIIRGWIFGTTLDSK  (NTD)  2:FPQSAPHGVVFLHVTYVPAQEKNFTTAPAICHDGKAHFPREGVFVSNGTHWFVTQRNFYEPQIITTDNTFVSGNC(CD)  3:TEVPVAIHADQLTPTWRVYSTGSNVFQTRAGCLIGAEHVNNSYECDIPIGAGICASYQT  4:SNFRVQPTESIVRFPNITNLCPFGEVFNATRFASVYAW(RBD) |
| 237 | 105.21 | 0.4317 | 1:QSAPHGVVFLHVTYVPAQEKNFTTAPAICHDGKAHFPREGVFVSNGTHWFVTQRNFYEPQIITTDNTFVSGNC(CD)  2:TEVPVAIHADQLTPTWRVYSTGSNVFQTRAGCLIGAEHVNNSYECDIPIGAGICAS  3:TQDLFLPFFSNVTWFHAIHVSGTNGTKRFDNPVLPFNDGVYFASTEKSNIIRGWIFGTTL(NTD)  4:KSFTVEKGIYQTSNFRVQPTESIVRFPNITNLCPFGEVFNATRFASVY(NTD,RBD) |
| 238 | 105.67 | 0.4313 | 1:QSAPHGVVFLHVTYVPAQEKNFTTAPAICHDGKAHFPREGVFVSNGTHWFVTQRNFYEPQIITTDNTFVSGNC(CD)  2:TEVPVAIHADQLTPTWRVYSTGSNVFQTRAGCLIGAEHVNNSYECDIPIGAGICAS  3:TQDLFLPFFSNVTWFHAIHVSGTNGTKRFDNPVLPFNDGVYFASTEKSNIIRGWIFGTTL(NTD)  4:KSFTVEKGIYQTSNFRVQPTESIVRFPNITNLCPFGEVFNATRFASVYA(NTD,RBD) |
| 239 | 106.13 | 0.4280 | 1:PQSAPHGVVFLHVTYVPAQEKNFTTAPAICHDGKAHFPREGVFVSNGTHWFVTQRNFYEPQIITTDNTFVSGNC(CD)  2:TEVPVAIHADQLTPTWRVYSTGSNVFQTRAGCLIGAEHVNNSYECDIPIGAGICAS  3:TQDLFLPFFSNVTWFHAIHVSGTNGTKRFDNPVLPFNDGVYFASTEKSNIIRGWIFGTTL(NTD)  4:KSFTVEKGIYQTSNFRVQPTESIVRFPNITNLCPFGEVFNATRFASVYA(NTD,RBD) |
| 240 | 106.59 | 0.4541 | 1:QSAPHGVVFLHVTYVPAQEKNFTTAPAICHDGKAHFPREGVFVSNGTHWFVTQRNFYEPQIITTDNTFVSGNC(CD)  2:TEVPVAIHADQLTPTWRVYSTGSNVFQTRAGCLIGAEHVNNSYECDIPIGAGICASYQTQ  3:DLFLPFFSNVTWFHAIHVSGTNGTKRFDNPVLPFNDGVYFASTEKSNIIRGWIFGTTL(NTD)  4:KSFTVEKGIYQTSNFRVQPTESIVRFPNITNLCPFGEVFNATRFASVYA(NTD,RBD) |
| 241 | 107.04 | 0.4508 | 1:PQSAPHGVVFLHVTYVPAQEKNFTTAPAICHDGKAHFPREGVFVSNGTHWFVTQRNFYEPQIITTDNTFVSGNC(CD)  2:TEVPVAIHADQLTPTWRVYSTGSNVFQTRAGCLIGAEHVNNSYECDIPIGAGICASYQTQ  3:DLFLPFFSNVTWFHAIHVSGTNGTKRFDNPVLPFNDGVYFASTEKSNIIRGWIFGTTL(NTD)  4:KSFTVEKGIYQTSNFRVQPTESIVRFPNITNLCPFGEVFNATRFASVYA(NTD,RBD) |
| 242 | 107.44 | 0.4432 | 1:PQSAPHGVVFLHVTYVPAQEKNFTTAPAICHDGKAHFPREGVFVSNGTHWFVTQRNFYEPQIITTDNTFVSGNC(CD)  2:TEVPVAIHADQLTPTWRVYSTGSNVFQTRAGCLIGAEHVNNSYECDIPIGAGICASYQTQ  3:DLFLPFFSNVTWFHAIHVSGTNGTKRFDNPVLPFNDGVYFASTEKSNIIRGWIFGTTL(NTD)  4:KSFTVEKGIYQTSNFRVQPTESIVRFPNITNLCPFGEVFNATRFASVYAW(NTD,RBD) |
| 243 | 107.81 | 0.4347 | 1:FPQSAPHGVVFLHVTYVPAQEKNFTTAPAICHDGKAHFPREGVFVSNGTHWFVTQRNFYEPQIITTDNTFVSGNC(CD)  2:TEVPVAIHADQLTPTWRVYSTGSNVFQTRAGCLIGAEHVNNSYECDIPIGAGICASYQTQ  3:DLFLPFFSNVTWFHAIHVSGTNGTKRFDNPVLPFNDGVYFASTEKSNIIRGWIFGTTL(NTD)  4:KSFTVEKGIYQTSNFRVQPTESIVRFPNITNLCPFGEVFNATRFASVYAW(NTD,RBD) |
| 244 | 108.08 | 0.4344 | 1:SFPQSAPHGVVFLHVTYVPAQEKNFTTAPAICHDGKAHFPREGVFVSNGTHWFVTQRNFYEPQIITTDNTFVSGNC(CD)  2:TEVPVAIHADQLTPTWRVYSTGSNVFQTRAGCLIGAEHVNNSYECDIPIGAGICASYQTQ  3:DLFLPFFSNVTWFHAIHVSGTNGTKRFDNPVLPFNDGVYFASTEKSNIIRGWIFGTTL(NTD)  4:KSFTVEKGIYQTSNFRVQPTESIVRFPNITNLCPFGEVFNATRFASVYAW(NTD,RBD) |
| 245 | 108.38 | 0.4498 | 1:QSAPHGVVFLHVTYVPAQEKNFTTAPAICHDGKAHFPREGVFVSNGTHWFVTQRNFYEPQIITTDNTFVSGNC(CD)  2:TEVPVAIHADQLTPTWRVYSTGSNVFQTRAGCLIGAEHVNNSYECDIPIGAGICASYQTQ  3:DLFLPFFSNVTWFHAIHVSGTNGTKRFDNPVLPFNDGVYFASTEKSNIIRGWIFGTTLDSKT  (NTD)  4:LKSFTVEKGIYQTSNFRVQPTESIVRFPNITNLCPFGEVFNATRFASVYA(NTD,RBD) |
| 246 | 108.83 | 0.4466 | 1:PQSAPHGVVFLHVTYVPAQEKNFTTAPAICHDGKAHFPREGVFVSNGTHWFVTQRNFYEPQIITTDNTFVSGNC(CD)  2:TEVPVAIHADQLTPTWRVYSTGSNVFQTRAGCLIGAEHVNNSYECDIPIGAGICASYQTQ  3:DLFLPFFSNVTWFHAIHVSGTNGTKRFDNPVLPFNDGVYFASTEKSNIIRGWIFGTTLDSKT  (NTD)  4:LKSFTVEKGIYQTSNFRVQPTESIVRFPNITNLCPFGEVFNATRFASVYA(NTD,RBD) |
| 247 | 109.24 | 0.4391 | 1:PQSAPHGVVFLHVTYVPAQEKNFTTAPAICHDGKAHFPREGVFVSNGTHWFVTQRNFYEPQIITTDNTFVSGNC(CD)  2:TEVPVAIHADQLTPTWRVYSTGSNVFQTRAGCLIGAEHVNNSYECDIPIGAGICASYQTQ  3:DLFLPFFSNVTWFHAIHVSGTNGTKRFDNPVLPFNDGVYFASTEKSNIIRGWIFGTTLDSKT  (NTD)  4:LKSFTVEKGIYQTSNFRVQPTESIVRFPNITNLCPFGEVFNATRFASVYAW(NTD,RBD) |
| 248 | 109.63 | 0.4189 | 1:STQDLFLPFFSNVTWFHAIHVSGTNGTKRFDNPVLPFNDGVYFASTEKSNIIRGWIFGTTLD  (NTD)  2:KVEAEVQIDRLITGRLQS(CH)  3:APHGVVFLHVTYVPAQEKNFTTAPAICHDGKAHFPREGVFVSNGTHWFVTQRNFYEPQIITTDNTFVSGNC(CD)  4:TEVPVAIHADQLTPTWRVYSTGSNVFQTRAGCLIGAEHVNNSYECDIPIGAGICASYQT  5:SNFRVQPTESIVRFPNITNLCPFGEVFNATRFASVYAW(RBD) |
| 249 | 110.09 | 0.4514 | 1:STQDLFLPFFSNVTWFHAIHVSGTNGTKRFDNPVLPFNDGVYFASTEKSNIIRGWIFGTTLDS  (NTD)  2:APHGVVFLHVTYVPAQEKNFTTAPAICHDGKAHFPREGVFVSNGTHWFVTQRNFYEPQIITTDNTFVSGNC(CD)  3:TEVPVAIHADQLTPTWRVYSTGSNVFQTRAGCLIGAEHVNNSYECDIPIGAGICASYQT  4:SNFRVQPTESIVRFPNITNLCPFGEVFNATRFASVYA(RBD)  5:DSFVIRGDEVRQIAPGQTG(RBD) |
| 250 | 110.56 | 0.4557 | 1:STQDLFLPFFSNVTWFHAIHVSGTNGTKRFDNPVLPFNDGVYFASTEKSNIIRGWIFGTTLDS  (NTD)  2:APHGVVFLHVTYVPAQEKNFTTAPAICHDGKAHFPREGVFVSNGTHWFVTQRNFYEPQIITTDNTFVSGNC(CD)  3:TEVPVAIHADQLTPTWRVYSTGSNVFQTRAGCLIGAEHVNNSYECDIPIGAGICASYQT  4:SNFRVQPTESIVRFPNITNLCPFGEVFNATRFASVYA(RBD)  5:DSFVIRGDEVRQIAPGQTGK(RBD) |
| 251 | 110.88 | 0.4508 | 1:STQDLFLPFFSNVTWFHAIHVSGTNGTKRFDNPVLPFNDGVYFASTEKSNIIRGWIFGTTLDS  (NTD)  2:APHGVVFLHVTYVPAQEKNFTTAPAICHDGKAHFPREGVFVSNGTHWFVTQRNFYEPQIITTDNTFVSGNC(CD)  3:TEVPVAIHADQLTPTWRVYSTGSNVFQTRAGCLIGAEHVNNSYECDIPIGAGICASYQT  4:SNFRVQPTESIVRFPNITNLCPFGEVFNATRFASVYA(RBD)  5:DSFVIRGDEVRQIAPGQTGKI(RBD) |
| 252 | 111.27 | 0.4552 | 1:STQDLFLPFFSNVTWFHAIHVSGTNGTKRFDNPVLPFNDGVYFASTEKSNIIRGWIFGTTLDSKTQS(NTD)  2:APHGVVFLHVTYVPAQEKNFTTAPAICHDGKAHFPREGVFVSNGTHWFVTQRNFYEPQIITTDNTFVSGNC(CD)  3:TEVPVAIHADQLTPTWRVYSTGSNVFQTRAGCLIGAEHVNNSYECDIPIGAGICASYQT  4:SNFRVQPTESIVRFPNITNLCPFGEVFNATRFASVYA(RBD)  5:DSFVIRGDEVRQIAPGQT(RBD) |
| 253 | 111.74 | 0.4722 | 1:STQDLFLPFFSNVTWFHAIHVSGTNGTKRFDNPVLPFNDGVYFASTEKSNIIRGWIFGTTLDSKTQS(NTD)  2:APHGVVFLHVTYVPAQEKNFTTAPAICHDGKAHFPREGVFVSNGTHWFVTQRNFYEPQIITTDNTFVSGNC(CD)  3:TEVPVAIHADQLTPTWRVYSTGSNVFQTRAGCLIGAEHVNNSYECDIPIGAGICASYQT  4:SNFRVQPTESIVRFPNITNLCPFGEVFNATRFASVYA(RBD)  5:DSFVIRGDEVRQIAPGQTG(RBD) |
| 254 | 112.21 | 0.4763 | 1:STQDLFLPFFSNVTWFHAIHVSGTNGTKRFDNPVLPFNDGVYFASTEKSNIIRGWIFGTTLDSKTQS(NTD)  2:APHGVVFLHVTYVPAQEKNFTTAPAICHDGKAHFPREGVFVSNGTHWFVTQRNFYEPQIITTDNTFVSGNC(CD)  3:TEVPVAIHADQLTPTWRVYSTGSNVFQTRAGCLIGAEHVNNSYECDIPIGAGICASYQT  4:SNFRVQPTESIVRFPNITNLCPFGEVFNATRFASVYA(RBD)  5:DSFVIRGDEVRQIAPGQTGK(RBD) |
| 255 | 112.61 | 0.5008 | 1:PQSAPHGVVFLHVTYVPAQEKNFTTAPAICHDGKAHFPREGVFVSNGTHWFVTQRNFYEPQIITTDNTFVSGNC(CD)  2:TEVPVAIHADQLTPTWRVYSTGSNVFQTRAGCLIGAEHVNNSYECDIPIGAGICASYQT  3:SNFRVQPTESIVRFPNITNLCPFGEVFNATRFASV(RBD)  4:AYSNNSIAIPTNFTISVTTEILPVS  5:TQDLFLPFFSNVTWFHAIHVSGTNGTKRFDNPVLPFNDGVYFASTEKSNIIRGWIFGTTLDS  (NTD) |
| 256 | 113.05 | 0.4735 | 1:PQSAPHGVVFLHVTYVPAQEKNFTTAPAICHDGKAHFPREGVFVSNGTHWFVTQRNFYEPQIITTDNTFVSGNC(CD)  2:TEVPVAIHADQLTPTWRVYSTGSNVFQTRAGCLIGAEHVNNSYECDIPIGAGICASYQT  3:SNFRVQPTESIVRFPNITNLCPFGEVFNATRFASVYA(RBD)  4:YSNNSIAIPTNFTISVTTEILPVS  5:TQDLFLPFFSNVTWFHAIHVSGTNGTKRFDNPVLPFNDGVYFASTEKSNIIRGWIFGTTLDS  (NTD) |
| 257 | 113.47 | 0.4898 | 1:PQSAPHGVVFLHVTYVPAQEKNFTTAPAICHDGKAHFPREGVFVSNGTHWFVTQRNFYEPQIITTDNTFVSGNC(CD)  2:TEVPVAIHADQLTPTWRVYSTGSNVFQTRAGCLIGAEHVNNSYECDIPIGAGICASYQT  3:SNFRVQPTESIVRFPNITNLCPFGEVFNATRFASVYA(RBD)  4:YSNNSIAIPTNFTISVTTEILPVS  5:TQDLFLPFFSNVTWFHAIHVSGTNGTKRFDNPVLPFNDGVYFASTEKSNIIRGWIFGTTLDSK  (NTD) |
| 258 | 113.93 | 0.4861 | 1:STQDLFLPFFSNVTWFHAIHVSGTNGTKRFDNPVLPFNDGVYFASTEKSNIIRGWIFGTTLDS  (NTD)  2:APHGVVFLHVTYVPAQEKNFTTAPAICHDGKAHFPREGVFVSNGTHWFVTQRNFYEPQIITTDNTFVSGNC(CD)  3:TEVPVAIHADQLTPTWRVYSTGSNVFQTRAGCLIGAEHVNNSYECDIPIGAGICASYQT  4:SNFRVQPTESIVRFPNITNLCPFGEVFNATRFASVYA(RBD)  5:DSFVIRGDEVRQIAPGQTGKIADYNYKL  (RBD) |
| 259 | 114.27 | 0.4262 | 1:STQDLFLPFFSNVTWFHAIHVSGTNGTKRFDNPVLPFNDGVYFASTEKSNIIRGWIFGTTLDSKTQSLLIVNNATNVVIKVCEFQFCNDP(NTD)  2:QSAPHGVVFLHVTYVPAQEKNFTTAPAICHDGKAHFPREGVFVSNGTHWFVTQRNFYEPQIITTDNTFVSGNC(CD)  3:TEVPVAIHADQLTPTWRVYSTGSNVFQTRAGCLIGAEHVNNSYECDIPIGAGICASYQT  4:SNFRVQPTESIVRFPNITNLCPFGEVFNATRFASVYA(RBD) |
| 260 | 114.74 | 0.4289 | 1:QSAPHGVVFLHVTYVPAQEKNFTTAPAICHDGKAHFPREGVFVSNGTHWFVTQRNFYEPQIITTDNTFVSGNC(CD)  2:TEVPVAIHADQLTPTWRVYSTGSNVFQTRAGCLIGAEHVNNSYECDIPIGAGICASYQT  3:SNFRVQPTESIVRFPNITNLCPFGEVFNATRFAS(RBD)  4:TQDLFLPFFSNVTWFHAIHVSGTNGTKRFDNPVLPFNDGVYFASTEKSNIIRGWIFGTTLDSKTQSLLIVNNATNVVIKVCEFQFCNDPFLGVY(NTD) |
| 261 | 115.26 | 0.4294 | 1:QSAPHGVVFLHVTYVPAQEKNFTTAPAICHDGKAHFPREGVFVSNGTHWFVTQRNFYEPQIITTDNTFVSGNC(CD)  2:TEVPVAIHADQLTPTWRVYSTGSNVFQTRAGCLIGAEHVNNSYECDIPIGAGICASYQT  3:SNFRVQPTESIVRFPNITNLCPFGEVFNATRFAS(RBD)  4:TQDLFLPFFSNVTWFHAIHVSGTNGTKRFDNPVLPFNDGVYFASTEKSNIIRGWIFGTTLDSKTQSLLIVNNATNVVIKVCEFQFCNDPFLGVYY(NTD) |
| 262 | 115.74 | 0.4288 | 1:QSAPHGVVFLHVTYVPAQEKNFTTAPAICHDGKAHFPREGVFVSNGTHWFVTQRNFYEPQIITTDNTFVSGNC(CD)  2:TEVPVAIHADQLTPTWRVYSTGSNVFQTRAGCLIGAEHVNNSYECDIPIGAGICASYQT  3:SNFRVQPTESIVRFPNITNLCPFGEVFNATRFAS(RBD)  4:TQDLFLPFFSNVTWFHAIHVSGTNGTKRFDNPVLPFNDGVYFASTEKSNIIRGWIFGTTLDSKTQSLLIVNNATNVVIKVCEFQFCNDPFLGVYYH  (NTD) |
| 263 | 116.26 | 0.4932 | 1:ALLAGTITSGWTFGAGAALQIPFAMQMAYRFNGIGVTQ(HR1)  2:DLFLPFFSNVTWFHAIHVSGTNGTKRFDNPVLPFNDGVYFASTEKSNIIRGWIFGTTLDS(NTD)  3:APHGVVFLHVTYVPAQEKNFTTAPAICHDGKAHFPREGVFVSNGTHWFVTQRNFYEPQIITTDNTFVSGNC(CD)  4:TEVPVAIHADQLTPTWRVYSTGSNVFQTRAGCLIGAEHVNNSYECDIPIGAGICASYQT  5:SNFRVQPTESIVRFPNITNLCPFGEVFNATRFASV(RBD) |
| 264 | 116.78 | 0.4847 | 1:ALLAGTITSGWTFGAGAALQIPFAMQMAYRFNGIGVTQ(HR1)  2:DLFLPFFSNVTWFHAIHVSGTNGTKRFDNPVLPFNDGVYFASTEKSNIIRGWIFGTTLDS(NTD)  3:APHGVVFLHVTYVPAQEKNFTTAPAICHDGKAHFPREGVFVSNGTHWFVTQRNFYEPQIITTDNTFVSGNC(CD)  4:TEVPVAIHADQLTPTWRVYSTGSNVFQTRAGCLIGAEHVNNSYECDIPIGAGICASYQT  5:SNFRVQPTESIVRFPNITNLCPFGEVFNATRFASVY(RBD) |
| 265 | 117.3 | 0.4819 | 1:SALLAGTITSGWTFGAGAALQIPFAMQMAYRFNGIGVTQ(HR1)  2:DLFLPFFSNVTWFHAIHVSGTNGTKRFDNPVLPFNDGVYFASTEKSNIIRGWIFGTTLDS(NTD)  3:APHGVVFLHVTYVPAQEKNFTTAPAICHDGKAHFPREGVFVSNGTHWFVTQRNFYEPQIITTDNTFVSGNC(CD)  4:TEVPVAIHADQLTPTWRVYSTGSNVFQTRAGCLIGAEHVNNSYECDIPIGAGICASYQT  5:SNFRVQPTESIVRFPNITNLCPFGEVFNATRFASVY(RBD) |
| 266 | 117.79 | 0.4823 | 1:QSAPHGVVFLHVTYVPAQEKNFTTAPAICHDGKAHFPREGVFVSNGTHWFVTQRNFYEPQIITTDNTFVSGNC(CD)  2:TEVPVAIHADQLTPTWRVYSTGSNVFQTRAGCLIGAEHVNNSYECDIPIGAGICASYQT  3:SNFRVQPTESIVRFPNITNLCPFGEVFNATRFASVYA(RBD)  4:LLAGTITSGWTFGAGAALQIPFAMQMAYRFNGIGVTQ(HR1)  5:DLFLPFFSNVTWFHAIHVSGTNGTKRFDNPVLPFNDGVYFASTEKSNIIRGWIFGTTLDS(NTD) |
| 267 | 118.24 | 0.4792 | 1:PQSAPHGVVFLHVTYVPAQEKNFTTAPAICHDGKAHFPREGVFVSNGTHWFVTQRNFYEPQIITTDNTFVSGNC(CD)  2:TEVPVAIHADQLTPTWRVYSTGSNVFQTRAGCLIGAEHVNNSYECDIPIGAGICASYQT  3:SNFRVQPTESIVRFPNITNLCPFGEVFNATRFASVYA(RBD)  4:LLAGTITSGWTFGAGAALQIPFAMQMAYRFNGIGVTQ(HR1)  5:DLFLPFFSNVTWFHAIHVSGTNGTKRFDNPVLPFNDGVYFASTEKSNIIRGWIFGTTLDS(NTD) |
| 268 | 118.74 | 0.4765 | 1:QSAPHGVVFLHVTYVPAQEKNFTTAPAICHDGKAHFPREGVFVSNGTHWFVTQRNFYEPQIITTDNTFVSGNC(CD)  2:TEVPVAIHADQLTPTWRVYSTGSNVFQTRAGCLIGAEHVNNSYECDIPIGAGICASYQT  3:SNFRVQPTESIVRFPNITNLCPFGEVFNATRFASVY(RBD)  4:TSALLAGTITSGWTFGAGAALQIPFAMQMAYRFNGIGVTQ(HR1)  5:DLFLPFFSNVTWFHAIHVSGTNGTKRFDNPVLPFNDGVYFASTEKSNIIRGWIFGTTLDS(NTD) |
| 269 | 119.19 | 0.4734 | 1:PQSAPHGVVFLHVTYVPAQEKNFTTAPAICHDGKAHFPREGVFVSNGTHWFVTQRNFYEPQIITTDNTFVSGNC(CD)  2:TEVPVAIHADQLTPTWRVYSTGSNVFQTRAGCLIGAEHVNNSYECDIPIGAGICASYQT  3:SNFRVQPTESIVRFPNITNLCPFGEVFNATRFASVY(RBD)  4:TSALLAGTITSGWTFGAGAALQIPFAMQMAYRFNGIGVTQ(HR1)  5:DLFLPFFSNVTWFHAIHVSGTNGTKRFDNPVLPFNDGVYFASTEKSNIIRGWIFGTTLDS(NTD) |
| 270 | 119.61 | 0.4889 | 1:PQSAPHGVVFLHVTYVPAQEKNFTTAPAICHDGKAHFPREGVFVSNGTHWFVTQRNFYEPQIITTDNTFVSGNC(CD)  2:TEVPVAIHADQLTPTWRVYSTGSNVFQTRAGCLIGAEHVNNSYECDIPIGAGICASYQT  3:SNFRVQPTESIVRFPNITNLCPFGEVFNATRFASVY(RBD)  4:TSALLAGTITSGWTFGAGAALQIPFAMQMAYRFNGIGVTQ(HR1)  5:DLFLPFFSNVTWFHAIHVSGTNGTKRFDNPVLPFNDGVYFASTEKSNIIRGWIFGTTLDSK(NTD) |
| 271 | 119.98 | 0.4812 | 1:FPQSAPHGVVFLHVTYVPAQEKNFTTAPAICHDGKAHFPREGVFVSNGTHWFVTQRNFYEPQIITTDNTFVSGNC(CD)  2:TEVPVAIHADQLTPTWRVYSTGSNVFQTRAGCLIGAEHVNNSYECDIPIGAGICASYQT  3:SNFRVQPTESIVRFPNITNLCPFGEVFNATRFASVY(RBD)  4:TSALLAGTITSGWTFGAGAALQIPFAMQMAYRFNGIGVTQ(HR1)  5:DLFLPFFSNVTWFHAIHVSGTNGTKRFDNPVLPFNDGVYFASTEKSNIIRGWIFGTTLDSK(NTD) |
| 272 | 120.35 | 0.4881 | 1:PQSAPHGVVFLHVTYVPAQEKNFTTAPAICHDGKAHFPREGVFVSNGTHWFVTQRNFYEPQIITTDNTFVSGNC(CD)  2:TEVPVAIHADQLTPTWRVYSTGSNVFQTRAGCLIGAEHVNNSYECDIPIGAGICASYQT  3:SNFRVQPTESIVRFPNITNLCPFGEVFNATRFASVYA(RBD)  4:QYTSALLAGTITSGWTFGAGAALQIPFAMQMAYRFNGIGVTQ(HR1)  5:DLFLPFFSNVTWFHAIHVSGTNGTKRFDNPVLPFNDGVYFASTEKSNIIRGWIFGTTLDS(NTD) |
| 273 | 120.77 | 0.5034 | 1:PQSAPHGVVFLHVTYVPAQEKNFTTAPAICHDGKAHFPREGVFVSNGTHWFVTQRNFYEPQIITTDNTFVSGNC(CD)  2:TEVPVAIHADQLTPTWRVYSTGSNVFQTRAGCLIGAEHVNNSYECDIPIGAGICASYQT  3:SNFRVQPTESIVRFPNITNLCPFGEVFNATRFASVYA(RBD)  4:QYTSALLAGTITSGWTFGAGAALQIPFAMQMAYRFNGIGVTQ(HR1)  5:DLFLPFFSNVTWFHAIHVSGTNGTKRFDNPVLPFNDGVYFASTEKSNIIRGWIFGTTLDSK(NTD) |
| 274 | 121.14 | 0.4957 | 1:FPQSAPHGVVFLHVTYVPAQEKNFTTAPAICHDGKAHFPREGVFVSNGTHWFVTQRNFYEPQIITTDNTFVSGNC(CD)  2:TEVPVAIHADQLTPTWRVYSTGSNVFQTRAGCLIGAEHVNNSYECDIPIGAGICASYQT  3:SNFRVQPTESIVRFPNITNLCPFGEVFNATRFASVYA(RBD)  4:QYTSALLAGTITSGWTFGAGAALQIPFAMQMAYRFNGIGVTQ(HR1)  5:DLFLPFFSNVTWFHAIHVSGTNGTKRFDNPVLPFNDGVYFASTEKSNIIRGWIFGTTLDSK(NTD) |
| 275 | 121.52 | 0.4998 | 1:QSAPHGVVFLHVTYVPAQEKNFTTAPAICHDGKAHFPREGVFVSNGTHWFVTQRNFYEPQIITTDNTFVSGNC(CD)  2:TEVPVAIHADQLTPTWRVYSTGSNVFQTRAGCLIGAEHVNNSYECDIPIGAGICAS  3:TQDLFLPFFSNVTWFHAIHVSGTNGTKRFDNPVLPFNDGVYFASTEKSNIIRGWIFGTTLDSK  (NTD)  4:FLPFQQFGRDIADTTDAVRDPQTLEILDITPCSFGGVSVITPGTNTSN  5:FRVQPTESIVRFPNITNLCPFGEVFNATRFASVYA(RBD) |
| 276 | 121.98 | 0.4967 | 1:PQSAPHGVVFLHVTYVPAQEKNFTTAPAICHDGKAHFPREGVFVSNGTHWFVTQRNFYEPQIITTDNTFVSGNC(CD)  2:TEVPVAIHADQLTPTWRVYSTGSNVFQTRAGCLIGAEHVNNSYECDIPIGAGICAS  3:TQDLFLPFFSNVTWFHAIHVSGTNGTKRFDNPVLPFNDGVYFASTEKSNIIRGWIFGTTLDSK  (NTD)  4:FLPFQQFGRDIADTTDAVRDPQTLEILDITPCSFGGVSVITPGTNTSN  5:FRVQPTESIVRFPNITNLCPFGEVFNATRFASVYA(RBD) |
| 277 | 122.44 | 0.5190 | 1:QSAPHGVVFLHVTYVPAQEKNFTTAPAICHDGKAHFPREGVFVSNGTHWFVTQRNFYEPQIITTDNTFVSGNC(CD)  2:TEVPVAIHADQLTPTWRVYSTGSNVFQTRAGCLIGAEHVNNSYECDIPIGAGICASYQTQ  3:DLFLPFFSNVTWFHAIHVSGTNGTKRFDNPVLPFNDGVYFASTEKSNIIRGWIFGTTLDSK(NTD)  4:FLPFQQFGRDIADTTDAVRDPQTLEILDITPCSFGGVSVITPGTNTSN  5:FRVQPTESIVRFPNITNLCPFGEVFNATRFASVYA(RBD) |
| 278 | 122.89 | 0.5159 | 1:PQSAPHGVVFLHVTYVPAQEKNFTTAPAICHDGKAHFPREGVFVSNGTHWFVTQRNFYEPQIITTDNTFVSGNC(CD)  2:TEVPVAIHADQLTPTWRVYSTGSNVFQTRAGCLIGAEHVNNSYECDIPIGAGICASYQTQ  3:DLFLPFFSNVTWFHAIHVSGTNGTKRFDNPVLPFNDGVYFASTEKSNIIRGWIFGTTLDSK(NTD)  4:FLPFQQFGRDIADTTDAVRDPQTLEILDITPCSFGGVSVITPGTNTSN  5:FRVQPTESIVRFPNITNLCPFGEVFNATRFASVYA(RBD) |
| 279 | 123.29 | 0.5091 | 1:PQSAPHGVVFLHVTYVPAQEKNFTTAPAICHDGKAHFPREGVFVSNGTHWFVTQRNFYEPQIITTDNTFVSGNC(CD)  2:TEVPVAIHADQLTPTWRVYSTGSNVFQTRAGCLIGAEHVNNSYECDIPIGAGICASYQTQ  3:DLFLPFFSNVTWFHAIHVSGTNGTKRFDNPVLPFNDGVYFASTEKSNIIRGWIFGTTLDSK(NTD)  4:FLPFQQFGRDIADTTDAVRDPQTLEILDITPCSFGGVSVITPGTNTSN  5:FRVQPTESIVRFPNITNLCPFGEVFNATRFASVYAW(RBD) |
| 280 | 123.68 | 0.4944 | 1:PQSAPHGVVFLHVTYVPAQEKNFTTAPAICHDGKAHFPREGVFVSNGTHWFVTQRNFYEPQIITTDNTFVSGNC(CD)  2:TEVPVAIHADQLTPTWRVYSTGSNVFQTRAGCLIGAEHVNNSYECDIPIGAGICASYQTQ  3:DLFLPFFSNVTWFHAIHVSGTNGTKRFDNPVLPFNDGVYFASTEKSNIIRGWIFGTTLDSK(NTD)  4:KFLPFQQFGRDIADTTDAVRDPQTLEILDITPCSFGGVSVITPGTNTSN  5:FRVQPTESIVRFPNITNLCPFGEVFNATRFASVYAW(RBD) |
